# Cognitive emotion regulation and personality: an analysis of individual differences in the neural and behavioral correlates of successful reappraisal

**DOI:** 10.1017/pen.2019.11

**Published:** 2019-11-07

**Authors:** Christoph Scheffel, Kersten Diers, Sabine Schönfeld, Burkhard Brocke, Alexander Strobel, Denise Dörfel

**Affiliations:** Differential and Personality Psychology, Faculty of Psychology, Technische Universität Dresden, Dresden, Germany

**Keywords:** personality, emotion regulation, fMRI, reappraisal, amygdala

## Abstract

A common and mostly effective emotion regulation strategy is reappraisal. During reappraisal, activity in cognitive control brain regions increases and activity in brain regions associated with emotion responding (e.g., the amygdala) diminishes. Immediately after reappraisal, it has been observed that activity in the amygdala increases again, which might reflect a paradoxical aftereffect. While there is extensive empirical evidence for these neural correlates of emotion regulation, only few studies targeted the association with individual differences in personality traits. The aim of this study is to investigate these associations more thoroughly. Seventy-six healthy participants completed measures of broad personality traits (Big Five, Positive and Negative Affect) as well as of more narrow traits (habitual use of emotion regulation) and performed an experimental fMRI reappraisal task. Participants were instructed to either permit their emotions or to detach themselves from the presented negative and neutral pictures. After each picture, a relaxation period was included. Reappraisal success was determined by arousal ratings and activity in the amygdala. During reappraisal, we found activation in the prefrontal cortex and deactivation in the left amygdala. During the relaxation period, an immediate aftereffect was found in occipital regions and marginally in the amygdala. Neither personality traits nor habitual use of emotion regulation predicted reappraisal success or the magnitude of the aftereffect. We replicated typical activation and deactivation patterns during intentional emotion regulation and partially replicated the immediate aftereffect in the amygdala. However, there was no association between personality traits and emotion regulation success.

Emotion regulation can be defined as any process by which individuals modify their emotional experiences, expressions, and physiology (Gross, 1998b), which enable them to function in their everyday lives. Deficits in emotion regulation may be associated with physical, as well as mental, health detriments (Davidson, Pizzagalli, Nitschke, & Putnam, 2002; Gross & Muñoz, 1995; Johnstone & Walter, 2014; Kanske, Heissler, Schönfelder, & Wessa, 2012). Gross (1998a) distinguishes between different emotion regulation strategies. The author assumes cognitive strategies such as Reappraisal, at an early stage of the emotion generation process, and response modulation such as expressive suppression late in the process (Gross, 1998a, 2014; Webb, Miles, & Sheeran, 2012). Reappraisal frequently has been shown to effectively reduce subjective arousal ratings, intuitive judgments, and self-reported negative affect (e.g., Feinberg, Willer, Antonenko, & John, 2012; Gross, 1998b; Ray, McRae, Ochsner, & Gross, 2010). Moreover, Aldao, Nolen-Hoeksema and Schweizer (2010) could meta-analytically show that more habitual use of Reappraisal is associated with less symptoms of psychopathology.

Intentional, cognitive emotion regulation in general is associated with an interplay of different brain regions. Consistently, activity in prefrontal and parietal regions involved in cognitive control and attention increases, while activation in areas associated with generating emotional responses is reduced (e.g., Buhle et al., 2014; Dörfel et al., 2014; Goldin, McRae, Ramel, & Gross, 2008; Kanske, Heissler, Schönfelder, Bongers, & Wessa, 2011; Kohn et al., 2014; McRae et al., 2010; Ochsner, Bunge, Gross, & Gabrieli, 2002; Phillips, Ladouceur, & Drevets, 2008; Walter et al., 2009). It is assumed that regions implicated in cognitive control exert an inhibitory effect on the amygdala via ventral prefrontal regions (Buhle et al., 2014; Lee, Heller, van Reekum, Nelson, & Davidson, 2012; Ochsner et al., 2002; Wager, Davidson, Hughes, Lindquist, & Ochsner, 2008).

## 1.1 Detachment as emotion regulation strategy

A specific Reappraisal strategy is detachment, which will be in focus of this investigation. By using detachment, one imagines to be an uninvolved observer thereby changing the perspective he or she is adopting toward the scenes or stimuli (Kalisch et al., 2005). Detachment has meta-analytically proved to be an effective emotion regulation strategy and superior over other forms of emotion regulation (Webb et al., 2012). With an effect size of *d*_+_ = 0.45, it had a small-to-medium-sized effect on outcomes in measures of emotion experience.

Similarly, by using detachment as an emotion regulation strategy, a reduction of amygdala activity as well as an activation in dorsolateral prefrontal cortex (dlPFC), inferior parietal cortex, and dorsal anterior cingulate cortex has been observed (Erk et al., 2010; Kalisch et al., 2005; Ochsner et al., 2002; Ochsner, Silvers, & Buhle, 2012; Walter et al., 2009). In comparison to other emotion regulation strategies, detachment increased activation specifically in the right angular gyrus (Dörfel et al., 2014).

## 1.2 Aftereffects in brain activity of emotion regulation

Gross (2014) stated that different strategies of emotion regulation have different short- and medium-term consequences. In line with this, immediate, short-term effects after detachment have been found (Walter et al., 2009). In a relaxation period after picture offset, hence after instructed down-regulation of negative emotions, activity in the amygdala increased and reached a peak. In contrast, after watching negative images with no regulation instruction, no such increase was observable. This could reflect an immediate, paradoxical aftereffect or a shift of amygdala activity due to detachment. Similar immediate aftereffects, for instance, were described in the context of thought suppression (Abramowitz, Tolin, & Street, 2001; Wegner, Schneider, Carter, & White, 1987). Interestingly, the extent of the immediate aftereffect in emotional reactivity in the Walter et al. (2009) study was positively related to thought suppression. In addition, such paradoxical effects were also observable in different brain regions (prefrontal and occipital regions) after emotion down-regulation and can be seen as task–rest interactions (Lamke et al., 2014). A medium-term regulation effect in a second task, several minutes after the regulation task, has also been shown in participants who were instructed to use detachment to regulate negative emotions (Erk et al., 2010; Walter et al., 2009). Furthermore, a positive association between the immediate aftereffect and the medium-term regulation effect was observable (Walter et al., 2009) pointing to a complex interplay between short-, middle-, and maybe even long-term effects of down-regulation of negative emotion.

## 1.3 Emotion regulation and individual differences

The experience and regulation of emotions can vary considerably across individuals (e.g., Gross & John, 2003; Mauss, Cook, Cheng, & Gross, 2007). Those variations have been associated with cognitive control processes (e.g., McRae, Jacobs, Ray, John, & Gross, 2012) and previous studies have shown that neuronal activity and connectivity during cognitive emotion regulation is associated with differences in emotion regulation success (Morawetz, Bode, Baudewig, & Heekeren, 2017) as well as with differences in habitual emotion regulation use (Drabant, McRae, Manuck, Hariri, & Gross, 2009; Vanderhasselt, Baeken, Van Schuerbeek, Luypaert, & De Raedt, 2013). Further, dysfunctions in emotion regulation are associated with mood and anxiety disorders (Davidson et al., 2002; Erk et al., 2010; Johnstone, van Reekum, Urry, Kalin, & Davidson, 2007; Johnstone & Walter, 2014). To gain a better understanding of individual differences in behavioral and neuronal emotion regulation patterns and their contribution to mental well-being, it seems informative to consider trait variables that may explain such individual differences. It is well known that Neuroticism increases the risk for several psychological disorders (e.g., depression) associated with deficient emotion regulation abilities (He, Song, Xiao, Cui, & McWhinnie, 2018; Hettema, Neale, Myers, Prescott, & Kendler, 2006; Kotov, Gamez, Schmidt, & Watson, 2010; Navrady et al., 2018). Further, the use of maladaptive emotion regulation strategies moderated the relationship between Neuroticism and depressive symptoms in one study (Yoon, Maltby, & Joormann, 2013). Individuals high on Neuroticism have been shown to experience more Negative Affect (Larsen & Ketelaar, 1991) and to have difficulties in disengaging attention from anxiety-provoking stimuli (Derryberry & Reed, 2002).

Associations between personality traits and the subjectively reported use of emotion regulation strategies, measured with the emotion regulation questionnaire (ERQ; Gross & John, 2003), are also well examined (Gross & John, 2003; John & Eng, 2014). Individuals with more negative affect, as measured with the Positive and Negative Affect Schedule (PANAS), show less habitual use of Reappraisal and more habitual use of suppression. Vice versa, more positive affect is associated with more habitual use of Reappraisal and less habitual use of Suppression. Therefore, individuals with more negative emotions might also show less emotion down-regulation success while using detachment.

Associations between the Big Five (Extraversion, Neuroticism, Openness, Agreeableness, Conscientiousness; McCrae & Costa, 2008) and emotion regulation have been reported, but there are mixed results. Most findings are concerned with Neuroticism and Extraversion. Neuroticism is related to less use of Reappraisal, more habitual use of Suppression, and emotion regulation strategies in general (John & Gross, 2004; Kokkonen & Pulkkinen, 2001). Extraversion is positively related to Positive Affect (Larsen & Ketelaar, 1991), *positive emotions* is even a facet of extraversion (McCrae & Costa, 2008), and habitual use of Reappraisal (Gross & John, 2003; John & Eng, 2014) as well as to understanding and regulation of emotions (as facets of emotional intelligence, Ciarrochi, Chan, & Caputi, 2000).

Evidence for associations between further personality dimensions and emotion regulation abilities exists, albeit less comprehensively. Individuals high in Agreeableness tend to put more effort into controlling their emotions (Tobin, Graziano, Vanman, & Tassinary, 2000). Openness and Conscientiousness (John, Naumann, & Soto, 2008) are known to have modest positive associations with habitual use of Reappraisal (Gross & John, 2003; John & Eng, 2014). With a hierarchical regression, Matsumoto (2006) was able to explain cultural differences in emotion regulation with Conscientiousness, Extraversion, and Neuroticism. Morawetz, Alexandrowicz and Heekeren (2017) predicted emotion regulation success with Neuroticism (successful increase), Conscientiousness (successful increase), and Openness (successful decrease) in a structural equation model.

Which leads to the following question: Are these modest relations of personality traits with behavioral emotion regulation success mirrored in individual differences at the neural level? Paschke et al. (2016) reported that trait self-control predicts emotion regulation success mirrored in amygdala down-regulation over a longer time-period. Harenski, Kim and Hamann (2009) found an association between prefrontal activity during emotion regulation and Neuroticism. Neuroticism also covaries with the volume of brain regions associated with negative affect, whereas Conscientiousness covaries with the volume of lateral prefrontal regions (DeYoung et al., 2010). In a fMRI study, individuals with higher Negative Affect tended to show greater activity in the amygdala during inspection of fearful images (Kret, Denollet, Grezes, & de Gelder, 2011). However, the number of studies showing convincing associations between individual differences in personality traits and behavioral and neural emotion regulation success is not satisfactory. Further research into this issue is urgently needed. Therefore, our aim in this investigation was to examine, whether personality traits, more specifically Positive and Negative Affect and the Big Five, have differential influence on emotion regulation success as measured by arousal ratings and amygdala activation during and after emotion regulation via detachment.

As a manipulation check, we needed to replicate findings of decreased arousal ratings as well as decreased activity in brain regions associated with negative emotions (e.g., amygdala) during down-regulation of negative emotions (behavioral and neuronal emotion regulation success, respectively), and increased activity in brain regions associated with cognitive control (e.g., dlPFC) during down-regulation of negative emotions. We needed to replicate the immediate paradoxical aftereffect in the amygdala. That means, permitting negative emotions during picture presentation leads to an increase in amygdala activation as compared to intentional emotion regulation, but after down-regulation of negative emotions (during the relax period), the amygdala is more strongly activated than after permitting emotions.

Considering associations between personality traits and emotion regulation success, we hypothesized that: (1) Self-reported Negative Affect, Neuroticism, and habitual use of Suppression are negatively associated with decrease in arousal ratings after emotion down-regulation, and Positive Affect, Extraversion, Openness, Agreeableness, Conscientiousness, and habitual use of Reappraisal are positively associated with decrease in arousal ratings after emotion down-regulation; (2) Self-reported Negative Affect, Neuroticism, and habitual use of Suppression are negatively associated with activation decreases in the amygdala during emotion down-regulation, and Positive Affect, Extraversion, Openness, Agreeableness, Conscientiousness, and habitual use of Reappraisal are positively associated with activation decreases in the amygdala during emotion down-regulation. Considering the immediate aftereffect in the amygdala wanted to explore (3) whether this effect is related to Positive Affect, Negative Affect, Neuroticism, Extraversion, Openness, Agreeableness, Conscientiousness, habitual use of Suppression or habitual use of Reappraisal.

## 2. Methods

In this analysis, we focus on individual differences in emotion regulation success. To address this issue, we therefore decided to combine two samples from two slightly different experiments from a greater study on the neural correlates and individual differences of emotion regulation and its aftereffects (SFB 940 Project A5). “We report how we determined our sample size, all data exclusions (if any), all manipulations, and all measures in the study” (Simmons, Nelson, & Simonsohn, 2012). Note that several results on other research questions of our study in sample 1 as well as sample 2 will be published elsewhere (Diers et al., in revision; Diers et al., in preparation).

### 2.1 Participants

Sample size calculation was done based on feasibility considerations. Thus, we aimed at a final sample size of over 48 participants per experiment. With the end of the data collection process, samples of the two experiments contained *N* = 47 participants each, mostly students, recruited from the university community (Diers et al., in revision). Nine participants had to be excluded due to missing of significant parts of the amygdala in fMRI images. Data of the remaining *N* = 85 healthy participants (36 male; age: 25 ± 4.4 years, range: 18–38) were analyzed. With respect to our focus on analyzing individual differences in emotion regulation success, we had a final sample size of *N* = 76. This sample size enabled us to detect correlations of *r* ≥ .39 with a power of 1-*β* = .80 and a Bonferroni-corrected two-tailed *α* = .00625 (because of eight personality measures in the study, see below 2.3.). All participants were right-handed and had no current or prior neurological or psychiatric illness or treatment. The local ethics committee of the Technische Universität Dresden approved the experimental protocol (reference number: EK 10012012). Participation was voluntary and written informed consent was obtained. Participants received financial compensation for their time and effort.

### 2.2 Experimental emotion regulation paradigm and procedure

Participants performed an emotion regulation task with negative (categories: animal, body, disaster, disgust, injury, suffering, violence, and weapons) and neutral (categories: objects, persons, and scenes) images. Pictures were taken from the International Affective Picture System (IAPS; Lang, Bradley, & Cuthbert, 2008) and the Emotional Picture Set (EmoPicS, Wessa et al., 2010) and divided into sets of 16 stimuli in order to have 1 picture sub-set for each condition (see below). Valence (V) and arousal (A) values of these sets are comparable to the ratings by Walter et al. (2009): For experiment one, two sets of negative pictures (set one: V = 2.71, A = 5.85; set two: V = 2.65, A = 5.69) and two sets of neutral images (set one: V = 5.17, A = 2.94; set two: V = 5.13, A = 2.96) were used. For experiment two, three sets of negative images (set one: V = 2.71, A = 5.85; set two: V = 2.65, A = 5.69; set three: V = 2.65, A = 5.55) and three sets of neutral images (set one: V = 5.17, A = 2.94; set two: V = 5.13, A = 2.96; set three: V = 5.19, A = 2.85) were used.

#### 2.2.1 Experiment 1

The measurement within the fMRI scanner lasted for approximately 60 min. It consisted of four experimental runs with a duration of 10 min each, an anatomical MRI measurement (6 min), and a re-exposure run (12 min). One week later, a second fMRI measurement was scheduled with a repetition of the re-exposure and a resting-state measurement. Given the scope of the present research questions, the same day re-exposure as well as the 1-week re-exposure and resting-state measurements will not be reported here.

During the four experimental runs, participants were asked to either permit or down-regulate their emotions. During the “permit” condition, participants should take a close look at the picture and permit any emotions that might arise. They were told to imagine immediately witnessing the depicted situation. However, they should not voluntary intensify their emotions, re-interpret the situation, or distract themselves. During the “detach” condition, they were asked to “take the position of a non-involved observer, thinking about the picture in a neutral way.” To achieve the detachment, participants were told to reduce personal involvement with the depicted situation, for example, by assuming personal or physical distance. Once more, participants were told not to re-interpret the situation as not real, attaching a different meaning to the situation, or distracting themselves. These strategies proved effectiveness in previous work (Diers, Weber, Brocke, Strobel, & Schönfeld, 2014; Dörfel et al., 2014; Paschke et al., 2016; Walter et al., 2009). All participants received written instructions including examples and completed a training session outside the MR scanner which took about 15 min and consisted of 16 trials. Following this, participants were interviewed about their emotion regulation strategies.

Each of the 4 runs of the main emotion regulation experiment consisted of 16 trials with 4 trials of each condition. In the first 10 s of each trial, participants viewed a picture. During the initial 2000 ms of this period, a semi-transparent overlay was presented across the center of the picture, which contained the instruction for each condition as a single word. For eight more seconds, only the picture was visible (Stimulation). Subsequently, participants should relax while viewing a fixation cross for 16–24 s (average: 20 s, Relaxation). Within this long period, BOLD response could return to its baseline level. The total duration of a single trial was 30 s, on average. After each run, retrospective arousal ratings were performed in the scanner, ranging from “not at all aroused” to “very highly aroused.” Participants rated their arousal for each condition.

#### 2.2.2 Experiment 2

The measurement within the fMRI scanner lasted for approximately 70 min. It consisted of four experimental runs with a duration of 11 min each, an anatomical MRI measurement (6 min), and a re-exposure run (12 min). One week later, a second fMRI measurement was scheduled with a repetition of the re-exposure and a resting-state measurement, which will not be reported here.

Participants were asked to permit, down-regulate, or intensify their emotions. Instructions for “permit” and “detach” condition conformed to the instructions given in experiment 1 (see above). During the “intensify” condition, participants were instructed to intensify their upcoming emotions by amplifying physical changes and imagining to participate in the depicted situation. Again, participants received written instructions including examples and completed a training session outside the MR scanner. It took about 15 min and consisted of 24 trials. The “intensify” condition will be neglected in this report (but see Diers et al., in preparation).

Each of the 4 runs of the main emotion regulation experiment consisted of 16 trials with 4 trials of each condition. In the first 8 s of each trial, participants viewed a picture. During the initial 2000 ms of this period, a semi-transparent overlay was presented across the center of the picture, which contained the instruction for each condition as a single word. For six more seconds, only the picture was visible (Stimulation). Subsequently, participants should relax while viewing a fixation cross for 12–20 s (average: 16 s, Relaxation). Within this long period, BOLD response could return to its baseline level. The total duration of a single trial was 24 s, on average. After each run, retrospective arousal ratings were performed in the scanner, ranging from “not at all aroused” to “very highly aroused.” Participants rated their arousal for each condition.

### 2.3 Psychometric measurements

After the scanner session, participants completed a variety of different questionnaires to measure personality traits, emotion regulation abilities, need for cognition, thought suppression, mindfulness, acceptance, worry, and anxiety. These questionnaires were the following: The German version of the revised NEO Five Factor Inventory (NEO-FFI; Costa & McCrae, 1992; German version, see: Borkenau & Ostendorf, 2007) assesses Neuroticism (internal consistency *α* = .85, retest-reliability *r*_*tt*_ = .80), Extraversion (*α* = .80, *r*_*tt*_ = .81), Openness (*α* = .71, *r*_*tt*_ = .76), Agreeableness (*α* = .71, *r*_*tt*_ = .65), and Conscientiousness (*α* = .85, *r*_*tt*_ = .81; Borkenau & Ostendorf, 2007). The PANAS (Watson, Clark, & Tellegen, 1988; German version: Janke & Glöckner-Rist, 2014) measures Positive Affect (PA), which is characterized by energy, concentration, and joyful commitment (α = .84, r_tt_ = .66, Krohne, Egloff, Kohlmann, & Tausch, 2016). Negative Affect (NA) is characterized by bad temper, tension, and anxiety (α = .86, r_tt_ = .54, Krohne et al., 2016). Habitual use of Suppression and habitual use of Reappraisal were assessed using the German version of the ERQ (Gross & John, 2003; German version: Abler & Kessler, 2009). Moreover, the personality trait need for cognition was assessed using the Need for Cognition Scale (NFC; Cacioppo & Petty, 1982; German version: Bless, Wanke, Bohner, Fellhauer, & Schwarz, 1994). As a measure for thought control, participants completed the White Bear Suppression Inventory (WBSI; Wegner & Zanakos, 1994; German version: Fehm, Höping, & Hoyer, 2002). Mindfulness- and acceptance-related questionnaires were the Mindful Attention Awareness Scale (MAAS; Brown & Ryan, 2003; German version: Michalak, Heidenreich, Strohle, & Nachtigall, 2008) and the Acceptance and Action Questionnaire (AAQ-II; Bond et al., 2011; German version: Hoyer & Gloster, 2013). As worry and anxiety related questionnaires, the Penn State Worry Questionnaire (PSWQ; Meyer, Miller, Metzger, & Borkovec, 1990; German version: Glöckner-Rist & Rist, 2014) and the State-Trait Anxiety Inventory (STAI; Spielberger, Gorsuch, & Lushene, 1970; German version: Grimm, 2009) were used. Finally, social desirability was assessed using the Social Desirability Scale (SES-17, Stöber, 1999).

The focus of the present investigation lies on the measurements of personality traits. Thus, out of the instruments above we analyzed the answers to the NEO-FFI, the PANAS, and the ERQ. Eight individuals had one omitted NEO-FFI item. Nevertheless, the scores of the related scales were computed according to recommendations of Borkenau and Ostendorf (2007). Entire questionnaires of *N* = 9 participants were completely missing, those were excluded from all analyses concerning personality traits.

### 2.4 Functional imaging

Imaging was performed on a 3.0 Tesla Siemens Magnetom Trio scanner (Siemens AG, Erlangen, Germany), using a 12 channel head coil. Functional data were obtained using a T2*-weighted echo-planar imaging sequence. The field of view (FOV) had a size of 192 mm × 192 mm, matrix size 64 × 64, flip angle 80°, slice gap 1 mm, repetition time (TR) = 2410 ms, and echo time (TE) = 25 ms. Forty-two axial slices were acquired with a voxel size of 3.0 mm × 3.0 mm × 2.0 mm. Stimuli in all sessions and the training were presented using Presentation (Neurobehavioral Systems, Albany, CA, USA). For each subject, anatomical (T1-weighted) images were acquired using an MPRAGE sequence consisting of 176 sagittal slices with a thickness of 1 mm (TR: 1900 ms, TE 2.26 ms, flip angle 9°, FOV: 256 mm × 256 mm, matrix size 256 × 256).

### 2.5 Data analysis

#### 2.5.1 Preprocessing of fMRI data

Preprocessing and statistical analysis were carried out using SPM 8 (http://www.fil.ion.ucl.ac.uk/spm/software/spm8), SPM 12 (http://www.fil.ion.ucl.ac.uk/spm/software/spm12), Matlab 2010b (MathWorks, Natick, MA), and R (https://www.r-project.org/). The first four volumes of each run were discarded. Preprocessing included motion correction, coregistration of individual functional and anatomical images, spatial normalization of the anatomical data to the MNI template, application of the estimated transformation parameters to the coregistered functional images using a resampling resolution of 2 × 2 × 2 mm³, and spatial smoothing of the functional images (FWHM 8 mm).

#### 2.5.2 First-level statistical analysis of fMRI data

For *experiment 1*, a general linear model (GLM) with regressors based on the experimental conditions, as well as six additional motion regressors of no interest, was used. Instructions and picture were set together as one event. For each condition, these events were modeled in two ways to cover transient and sustained responses. Temporal patterns were modeled as stick function (0 s duration, transient responses) or as boxcar function (10 s duration, sustained responses, respectively). Results will mainly be described for boxcar function. Results for the stick function model will be reported in the Supplementary Material. According to previous analyses about “rest” periods (Lamke et al., 2014), post-regulation phase (Relaxation) was also included in our model. Starting point was the picture offset and it lasted as long as the Stimulation period (10 s). This resulted in eight regressors of interest (“Neutral Permit,” “Neutral Detach,” “Negative Permit,” and “Negative Detach” for Stimulation and Relaxation period, respectively). All regressors were convolved with the canonical hemodynamic response function (HRF). The four runs of the imaging experiment were combined within one fixed-effects model.

The GLM for *experiment 2* corresponded to the GLM for experiment 1, except it yielded in 12 regressors of interest (“Neutral Permit,” “Neutral Detach,” “Neutral Intensify,” “Negative Permit,” “Negative Detach,” “Negative Intensify” each for Stimulation and Relaxation period). As described above, the intensify condition will be neglected. The duration of the regressors was 0 s (for stick function) and 8 s (for boxcar function), respectively.

#### 2.5.3 Second-level statistical analysis of fMRI data

As a manipulation check, the contrasts “Negative Permit Stimulation” > “Negative Detach Stimulation” (Permitting) and “Negative Detach Stimulation” > “Negative Permit Stimulation” (Detachment) were analyzed using second-level one-sample t-tests. Therefore, respective contrast images from the first level of both experiments were entered in the analysis. Results of the *T* – statistics were thresholded at *p* < .05 family wise error (FWE, Nichols & Hayasaka, 2003) corrected.

Our analysis of the *immediate aftereffect* was based on the procedure used by Lamke et al. (2014). On the first level, reverse task rest interactions were computed with the contrast [(“Negative Permit Stimulation” > “Negative Detach Stimulation”) > (“Negative Detach Relaxation” > “Negative Permit Relaxation”)]. These first-level contrasts of both experiments were entered into a second-level one-sample t-test to test for significant interaction effects in different brain regions. Interaction effects for one region are only interpretable, if one region showing this effect also shows significant activation in the second-level one-sample t-tests of the stimulation and relaxation contrasts (Lamke et al., 2014). Therefore, results of the interaction effect were inclusively masked with the two contrasts “Negative Permit Stimulation” > “Negative Detach Stimulation” and “Negative Detach Relaxation” > “Negative Permit Relaxation.” The *extent of the immediate aftereffect* was determined by extracting contrast estimates for the contrast “Negative Detach Relaxation” > “Negative Permit Relaxation” in our regions of interest (ROIs). Contrast estimates for relevant contrasts were extracted and computed using the SPM summarize function. Activation time courses in our ROIs were extracted using rfxplot toolbox (Glascher, 2009) (http://rfxplot.sourceforge.net).

For all second-level contrasts, the number of experiment (one or two) was entered as covariate, consequently controlling for variance resulting from this variable.

#### 2.5.4 ROI-analysis

We defined the ROIs using second-level peak activity in bilateral amygdala during the main effect of emotion (contrast: “Negative Stimulation” > “Neutral Stimulation”). Around each of the two voxels (left: *x* = −22, *y* = −6, *z* = −14; right: *x* = 22, *y* = −6, *z* = −12) a sphere was built (radius: 3 mm) and mean activity within these spheres for the contrasts of interest (see below) extracted for each participant using MarsBaR (http://marsbar.sourceforge.net/). Peak voxel for transient responses slightly differed (left: *x* = −18, *y* = −6, *z* = −14; right: *x* = 22, *y* = −6, *z* = −12).

#### 2.5.5 Association between behavioral and neuronal emotion regulation success

After computing the mean ratings of self-reported arousal after each run we determined *behavioral emotion regulation success* as the difference between arousal ratings for Negative Permit and Negative Detach. *Neuronal emotion regulation success* was defined as activity in the aforementioned amygdala ROIs for the contrast “Negative Permit Stimulation” > “Negative Detach Stimulation.”

To check, whether changes in neural activation during emotion down-regulation are correlated to self-reported emotional arousal, behavioral and neuronal emotion regulation success were entered into a partial correlation analysis with information about the experiment (1 or 2) as covariate.

#### 2.5.6 Hypotheses testing

To test for inter-individual differences in *behavioral emotion regulation success*, a hierarchical regression analysis was performed. In the first step, experiment as a covariate was entered, because the design of the tasks in the two experiments slightly differed which could have influenced ER success. In the second step, we entered PA, NA (as measurements for Positive and Negative Affect), Extraversion, and Neuroticism, as well as habitual use of Reappraisal and Suppression. We entered those predictors in one step, because of previous findings of associations between them. In the third step, Openness, Agreeableness, and Conscientiousness (as additional personality traits) were added. Data of *N* = 9 participants with completely missing questionnaires were excluded from this analysis. Arousal ratings of *N* = 5 more participants were also missing, which added up to *N* = 71 data sets entered in the analysis. Regression coefficients were considered significant if *p*_*corr*_ < .0056 (because of nine correlations testing our hypothesis).

Subsequently, to test for individual differences in *neuronal emotion regulation success*, a hierarchical regression was conducted. Similar to the hierarchical regression for behavioral emotion regulation success, experiment was entered first, PA, NA, Extraversion, and Neuroticism, as well as habitual use of Reappraisal and Suppression were entered second, and Openness, Agreeableness, and Conscientiousness as the third step. Again, data of *N* = 9 participants with completely missing questionnaires were excluded in this analysis. Statistical significance for these computations was *p*_*corr*_ < .0056.

To analyze individual differences personality and emotion regulation aftereffects, the extent of the *immediate aftereffect* in the aforementioned amygdala ROIs together with PA, NA, Extraversion, Neuroticism, Openness, Agreeableness, Conscientiousness, habitual use of Reappraisal and Suppression was entered in a partial correlation analysis, as well as experiment as covariate. Once again, only data of participants with complete questionnaire scores were included in the analysis.

## 3. Results

The data and R code used for computations and statistical analyses are available at https://osf.io/fn9d3/.

### 3.1 Behavioral and neuronal effects of emotion regulation (manipulation check)

#### 3.1.1 Behavioral and neuronal emotion regulation success

On average, participants showed significantly lower self-reported arousal after detachment from negative pictures *M* = 16, *SD* = 63.9, compared to permitting emotions *M* = −0.3, *SD* = 66.1, *V* = 2391, *p* < .001, with a moderate-to-large effect (*r* = .48). Detachment from negative pictures yielded significant activation in prefrontal regions as compared to permitting emotions. Permitting emotions yielded significant activation in occipital regions. This effect had a size of *d* = 1.1885. The ROI-analysis showed activation in the left amygdala, but not in the right amygdala for the contrast “Negative Permit Stimulation > Negative Detach Stimulation” (neuronal emotion regulation success). Detailed information about neuronal effects of ER can be found in Supplementary Material 2.^1^

To compare behavioral and neuronal ER success, Spearman correlations were computed. Arousal ratings were not related to left and right amygdala activity during emotion regulation (all *p* > .05, see Supplementary Material 3).

#### 3.1.2 Immediate aftereffect in the amygdala

**Figure 1.**
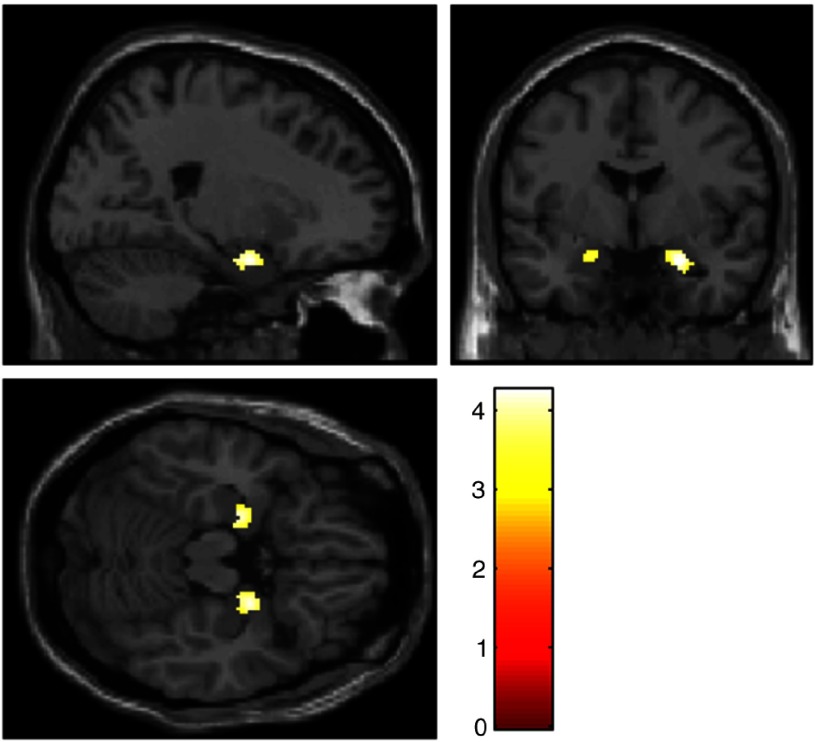
Amygdala activation for the contrast “(‘Negative Permit Stimulation’ > ‘Negative Detach Stimulation’) > (‘Negative Detach Relaxation’ > ‘Negative Permit Relaxation’)”, *p* < .001 uncorr. Slices are at *x* = 22 (top left), *y* = −2.9 (top right), *z* = −18.4 (bottom left).

A ROI-analysis revealed significant activation in the left (*x* = −22, *y* = −10, *z* = −20, *T* = 3.38, *p*_*FWE*_ = .026) and right (*x* = 22, *y* = −8, *z* = −22, *T* = 3.79, *p*_*FWE*_ = .007) amygdala for the contrast “Negative Detach Relaxation > Negative Permit Relaxation.” Even a significant interaction effect was observable for parts of the amygdala (left: *x* = −20, *y* = −10, *z* = −20, *T* = 4.14, *p*_*FWE*_ = .004; right: *x* = 24, *y* = −2, *z* = −22, *T* = 4.26, *p*_*FWE*_ = .003), but also for different brain regions including occipital regions and prefrontal regions (see Figure 1, for details please see Supplementary Material 4). After applying masking to this result, only occipital activation remained as a significant *reverse task–rest interaction* (for detailed results of sustained and transient responses, see Supplementary Material 5).

**Figure 2.**
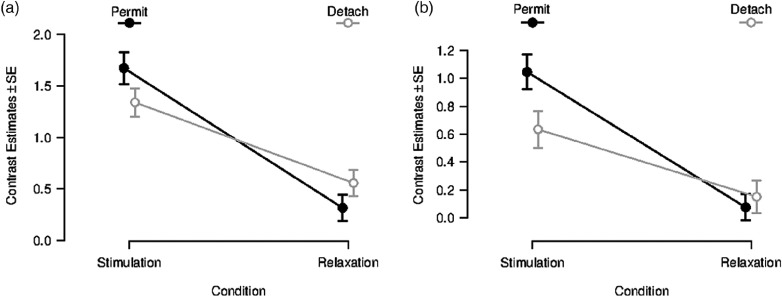
Contrast estimates for sustained responses in a functionally defined ROI in the left amygdala (*x* = −22, *y* = −6, *z* = −14). Left (a): Beta-values during and after inspection of negative images for the conditions “permit” (black line) and “detach” (gray line); error bars indicate *SD*. Right (b): Beta-values during and after inspection of neutral images for the conditions “permit” (black line) and “detach” (gray line); error bars indicate *SD*.

After masking the interaction effect, no reverse task–rest interaction effect was observable in the amygdala. This led to a more detailed statistical inspection of the *contrast estimates* in our functionally defined amygdala ROIs for different conditions (see Figure 2 for left amygdala, for right amygdala see Supplementary Material 7, Figure S7-1). In the left amygdala, no significant immediate aftereffect was observable (Figure 2a). Moreover, a similar pattern of contrast estimates was observable for the Neutral Stimulation and Neutral Relaxation period. Equal patterns are noticeable for contrast estimates in the right amygdala ROI (see Supplementary Material 7). Contrast estimates for left and right amygdala ROI for transient responses can be found in Supplementary Material 7.

**Figure 3.**
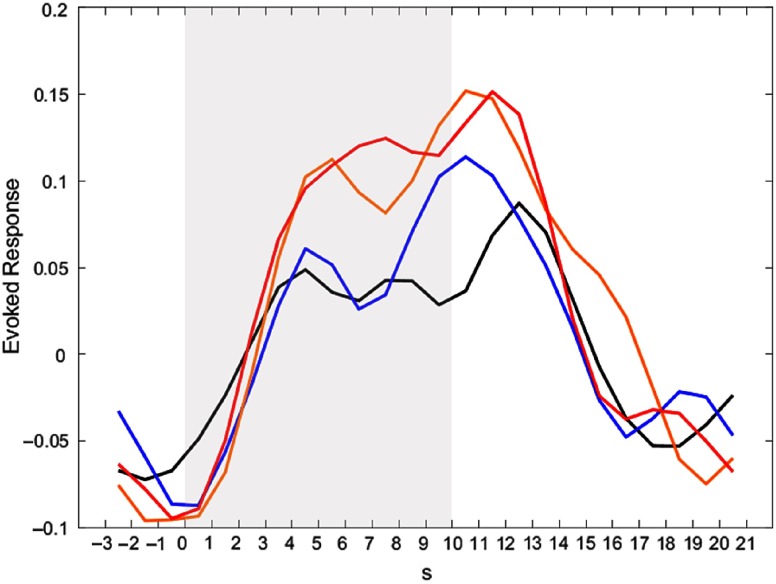
Activation time courses in the left amygdala for different conditions. Peak voxel was *x* = −22, *y* = −6, *z* = −14. Color indicates different conditions: black – neutral detach, blue – neutral permit, orange – negative detach, red – negative permit. Shaded area indicates phase of picture presentation.

To get a better impression of the *temporal course* of activation in the amygdala, post-hoc analyses for our ROIs were conducted (see Figure 3 for the activation time course in the left amygdala; the activation time course of the right amygdala can be found in Supplementary Material 8). In all conditions, activation in the amygdala rises during Stimulation. It is noteworthy that in the Detach Stimulation condition activation in the amygdala shows a reduction. Activation during “Neutral Stimulation” is notably lower than during “Negative Stimulation.” Then, amygdala activation rises again and finds its peak right after picture offset, during Relaxation.

### 3.2 Emotion regulation success and personality traits

#### 3.2.1 Behavioral emotion regulation success and personality traits

To investigate the effect of personality traits on behavioral emotion regulation success, a hierarchical regression was computed with first, sample, second PA, NA, Extraversion, Neuroticism, habitual use of Reappraisal and Suppression, and third Openness, Agreeableness, and Conscientiousness as predictors. No variable predicted changes in ER success after adjusting the α-level for multiple comparisons (*p*_*corr*_ < .0056). Covariate sample showed a trend toward significance at the unadjusted α-level (*t* = −1.94, *p* = .057), indicating less ER success in sample 2. Overall, the fit of this extended model was relatively low with *R²* = .16 (*p* = .347) and was not superior to the reduced model (*ΔR²* = .057, *F*_(df1,df2)_ = 1.35, *p* = .267, see Table 1).

**Table 1.** Summary of hierarchical regression analysis for variables predicting behavioral emotion regulation success

Variable	β	*t*	*p*	*R*	*R²*	*ΔR²*
Step 1				.24	.058	.058
Sample	−.24	−2.16	.034			
Step 2				.32	.103	.045
Sample	−.22	−1.77	.082			
Negative affect	−.02	−0.11	.912			
Positive affect	.04	0.21	.832			
Neuroticism	.12	0.65	.521			
Extraversion	.24	1.28	.206			
ERQ – Suppression	.06	0.46	.650			
ERQ – Reappraisal	−.11	−0.83	.412			
Step 3				.40	.160	.057
Sample	−.25	−1.94	.057			
Negative affect	−.07	−0.46	.646			
Positive affect	.14	0.77	.447			
Neuroticism	.19	1.02	.313			
Extraversion	.26	1.37	.175			
ERQ – Suppression	−.03	−0.22	.828			
ERQ – Reappraisal	−.07	−0.48	.635			
Openness	−.03	−0.24	.808			
Agreeableness	−.29	−1.99	.051			
Conscientiousness	−.08	−0.51	.610			

*Note. N* = 71.

#### 3.2.2 Neuronal emotion regulation success and personality traits

To investigate the effect of personality traits on neuronal emotion regulation success, a hierarchical regression was performed with first, sample, second PA, NA, Extraversion, Neuroticism, habitual use of Reappraisal and Suppression, and third Openness, Agreeableness, and Conscientiousness as predictors. Only results for left amygdala will be presented here, for results for the right amygdala please see Supplementary Material 3. Sample did not explain variance in neuronal ER success (see Table 2). In the second step, PA, NA, Extraversion, Neuroticism, habitual use of Reappraisal and Suppression (ERQ) were added. At the nominal level of significance, ERQ-Reappraisal showed a significant positive association (*t* = 2.14, *p* = .036). In the third step (Openness, Agreeableness, and Conscientiousness), Openness showed a significant negative association with amygdala activation (*t* = −2.03, *p* = .046). However, these effects did not survive correction for multiple comparisons *(p*_*corr*_ < .0056). Quality of the extended regression model was relatively low with *R²* = .142 (*p* = .395) and was not superior to the reduced model (*ΔR²* = .058, *F*_(df1,df2)_ =1.45, *p* = .236).

**Table 2.** Summary of hierarchical regression analysis for variables predicting neuronal emotion regulation success (left amygdala activity of sustained responses during emotion regulation)

Variable	β	*t*	*p*	*R*	*R²*	*ΔR²*
Step 1				.06	.004	.004
Sample	.06	0.57	.570			
Step 2				.29	.084	.080
Sample	−.10	−0.79	.434			
Negative affect	−.09	−0.60	.550			
Positive affect	−.10	−0.59	.557			
Neuroticism	−.13	−0.74	.461			
Extraversion	−.14	−0.76	.449			
ERQ – Suppression	.00	0.00	.996			
ERQ – Reappraisal	.24	1.79	.078			
Step 3				.38	.142	.058
Sample	−.12	−0.94	.353			
Positive affect	−.06	−0.40	.689			
Negative affect	−.08	−0.41	.685			
Neuroticism	−.11	−0.62	.538			
Extraversion	−.14	−0.73	.471			
ERQ – Suppression	−.03	−0.18	.856			
ERQ – Reappraisal	.30	2.14	.036			
Openness	−.25	−2.03	.046			
Agreeableness	−.05	−0.34	.731			
Conscientiousness	.01	0.08	.934			

*Note. N* = 76.

For results of hierarchical regression analysis predicting right amygdala activity and analyses concerning transient responses, please see Supplementary Material 6.

### 3.3 Association of the immediate aftereffect with personality traits

To explore, whether the immediate aftereffect was related to personality traits, a partial correlation analysis was computed controlling for experiment (1 or 2). Again, not all variables were normal distributed (see Supplementary Material 1). Therefore, a partial Spearman correlation was computed. We did not find any significant associations between the magnitude of the aftereffect in the amygdala and Positive and Negative Affect, the Big Five personality traits, or habitual use of Reappraisal and Suppression (p > .05). For detailed results, please see Supplementary Material 9.

## 4. Discussion

In the present study, we investigated whether inter-individual differences in personality traits are related to differences in neural activity during emotion regulation using detachment from negative stimuli and permission of negative emotions. As a prerequisite, down-regulation of negative emotions was successful in the combined sample, as indicated by significantly lower subjective arousal ratings, as well as by down-regulation of the amygdala and occipital regions. In contrast, prefrontal areas were stronger activated during emotion regulation as compared to permitting one’s emotions. Against our hypotheses, personality traits did not predict differences in subjective arousal ratings, as well as activity in the amygdala during emotion regulation. A further aim of this study was to investigate associations between an immediate aftereffect in the amygdalae after picture offset Walter et al. (2009) and personality traits. Immediate aftereffects could be found in different brain regions including the amygdala. However, after applying a stringent statistical correction, only effects in occipital and temporal regions remained. Personality traits were not related to the immediate amygdala aftereffect.

### 4.1 Association between personality and individual differences in behavioral and neuronal emotion regulation success

Neither the personality dimensions of the Big Five, nor Positive and Negative Affect nor the habitual use of Reappraisal and Suppression could explain variance in behavioral emotion regulation success measured via arousal ratings, as well as in neural emotion regulation success. These findings do not fit with the existing literature (e.g., Drabant et al., 2009; Gross & John, 2003; John & Gross, 2004; Morawetz, Bode et al., 2017), possible reasons for divergent results will be discussed in the following section.

Especially for Neuroticism and Negative Affect, there is strong evidence indicating limited emotion regulation abilities (Derryberry & Reed, 2002; Gross & John, 2003; Kokkonen & Pulkkinen, 2001). Gross and John (2003) found that Neuroticism is moderately associated with less habitual use of Reappraisal and more habitual use of Suppression. According to these results, we inferred that persons with higher Neuroticism show less emotion regulation success when using detachment, a form of Reappraisal. However, ratings could be affected by memory processes and differ from an immediate assessment because arousal ratings were assessed retrospectively after each block. The associations between personality dimensions and subjective evaluation of emotion regulation success remain therefore unclear. Additionally, associations between personality traits and habitual use of Reappraisal measured by self-report questionnaire are modest (Gross & John, 2003). It has been discussed that questionnaire measurements relate to actual behavior only to a low degree (Back, Schmukle, & Egloff, 2009; Mischel, 1968). We assume that our measurement (actual behavior during an emotion regulation task in the scanner instead of self-reports) might explain the diminution of the previously reported modest associations between personality traits and emotion regulation. Especially for personality traits (measured via questionnaire, e.g., Conscientiousness, Openness, John & Eng, 2014), where rather small correlations with habitual use of Reappraisal have been reported, it is more likely to find insignificant effects. To overcome this problem, it would be of advantage to examine larger samples to have sufficient number of cases in each subgroup. By doing so it should be possible to find at least modest associations between Neuroticism and Conscientiousness with behavioral emotion regulation success in Reappraisal tasks (Morawetz, Alexandrowicz et al., 2017). However, the analyses by Morawetz, Alexandrowicz et al. (2017) differ from our analyses in several ways. First, Morawetz et al. used structural equation modeling to predict behavioral emotion regulation success, integrating the subscales of several personality measures as latent factors and individual factor score vectors (resulting from modeling the items of each measure as manifest variables). Second, the final model not only contained the personality measures, but also inferior frontal gyrus and amygdala activity during the emotion regulation task to predict behavioral emotion regulation success. Instead of using amygdala activation as a dependent variable, Morawetz et al. defined it as a predictor. In contrast, based on previous studies of our work group and others (Diers et al., 2014; Dörfel et al., 2014; Ochsner et al., 2004; Paschke et al., 2016; Walter et al., 2009) we decided that emotion regulation success can be measured by amygdala down-regulation as well as by subjective arousal ratings.

Although some studies found associations between brain activity and personality traits (e.g., Harenski et al., 2009), our null finding is in accordance with other results reported in the literature. For example, during processing of negative emotional facial expression, frontal activity and activity in the amygdala was predicted by everyday Reappraisal use, but not by personality traits (Drabant et al., 2009). In line with this, we found a small, positive association (*β* = .30) between the habitual use of Reappraisal and activity in the left amygdala during intentional emotion regulation on the nominal level of significance. However, this result did not survive correction for multiple comparisons.

#### 4.1.1 Limitations of the measurement methods

Additionally, we assume that broad personality constructs rather show associations with patterns of emotion regulation strategy implementation across different situations (emotion regulation flexibility, see Aldao, Sheppes, & Gross, 2015; Dore, Silvers, & Ochsner, 2016) instead of emotion regulation success using only a single strategy. For instance, it has been reported that individuals high in Neuroticism prefer to increase (instead of decreasing) the experience of worry prior to taking a difficult test (Tamir, 2005). Neuroticism has also been shown to be negatively related to psychological flexibility, while Conscientiousness showed a positive association with flexibility (Latzman & Masuda, 2013). However, no study so far focused on individual differences in the flexible implementation of different emotion regulation strategies and its interaction with personality dispositions (Kobylinska & Kusev, 2019).

A related problem might be the different hierarchical level of our measures. With personality traits, we investigated broad constructs for the prediction of a very narrow criterion, that is, the subjective arousal rating and the amygdala activation after viewing negative pictures. If we follow the Brunswik Symmetry concept, “predictors and criteria have to be symmetrical to one another to obtain maximum predictability” (Wittmann & Süß, 1999, p. 79). In this case, the habitual use of Reappraisal and personality traits such as Neuroticism reveal more symmetry, making it more likely to discover significant associations. However, there has also been critique about the association between the very broad constructs of the Big Five and the narrower (personality) construct of habitual use of emotion regulation (John & Eng, 2014). One solution for both problems would be the alignment of hierarchical levels on the predictor and the criterion side. Concerning our study, the next step would be to choose more narrow personality facets as predictors, such as Anxiety, Depressivity, and Emotional Volatility (which constitute Neuroticism on a higher level, see Soto & John, 2017).

Psychological disorders have shown to be associated with impairments in emotion regulation abilities (Davidson et al., 2002; Erk et al., 2010; Johnstone et al., 2007; Johnstone & Walter, 2014). These disorders are also associated with Neuroticism (Hettema et al., 2006; Kotov et al., 2010). In terms of depression, Yoon et al. (2013) could show that the relation between Neuroticism and severity of depressive symptoms is fully mediated by use of maladaptive emotion regulation strategies. Perhaps, variance in emotion regulation abilities in healthy individuals is limited and therefore hard to detect with rather small sample sizes. To provide increased variance in these abilities, one could rather focus on comparisons between healthy individuals and individuals with the aforementioned disorders. Additionally, it would be of great interest for future investigations, whether type, amount, or severity of symptoms of other psychological disorders also can be explained by individual differences in the use of emotion regulation strategies rather than Neuroticism.

#### 4.1.2 The impact of situational strength

A further explanation for our null findings could be the situational strength realized by the experimental setting in our study (Mischel, 1968; Snyder & Ickes, 1985). Situational strength is defined as cues within a situation that maximize (in weak situations) or minimize (in strong situations) individual differences in personality. In our case, participants were exposed to very negative pictures (valence: 2.65–2.71; arousal: 5.55–5.85) within the MRI scanner, while instructed to engage in volitional emotion regulation. We chose highly negative stimuli in order to ensure reliable activation of brain structures for emotional processing. This could have resulted in a strong situation, where differences in down-regulation of negative emotions could not be explained by individual differences in personality traits. This would imply that emotion regulation of neutral pictures should act as a weak situation and therefore emphasize individual differences. However, a brief visual inspection of activation patterns of the contrasts “Neutral Detach Stimulation” > “Neutral Permit Stimulation” and “Neutral Permit Stimulation” > “Neutral Detach Stimulation” showed patterns similar to negative conditions. The latter contrast revealed a significant amygdala activation on FWE level (small volume correction). Subsequent analyses showed that changes in arousal ratings after detachment during inspection of neutral pictures could not be predicted by personality traits. Also, personality traits could not predict individual differences in amygdala activation during regulation of neutral pictures.

Individual differences in personality traits also failed to explain variance in the neural aftereffects of volitional emotion regulation. Besides the already mentioned symmetry problems between our predictors and criteria, this also might be explained by the specific data analysis procedure we choose to investigate the aftereffects. By modeling the individual responses to the experimental conditions as sustained responses (using boxcar functions), we might have missed the more transient responses in the respective brain areas (for further differences between transient and sustained responses, please see Diers et al., in revision). Additionally, a very stringent statistical masking procedure was used that led to a disappearance of the aftereffect in the amygdala. In further investigations, it could be examined, whether different approaches and methods alter associations between personality traits and the immediate aftereffect.

## 5. Limitations

Results regarding arousal ratings are limited, because these ratings were recorded retrospectively after each block. As we purported to replicate findings of Walter et al. (2009) regarding the immediate aftereffect, we aligned our experimental design on the design of their study. Walter et al. (2009) argued that an arousal rating after picture offset in the relaxation period would alter time courses of the HRF (see also Burklund, Creswell, Irwin, & Lieberman, 2014). Thus, self-evaluating cognitive processes could not interfere with this interval. However, retrospective arousal ratings could be more affected by memory processes and therefore differ from an immediate assessment. The impact on associations between personality dimensions and arousal ratings is unclear. Maybe, the evaluation of an arousal is related to personality, but memory effects obscure this association. In our study, no association between amygdala activity and subjective arousal ratings was observable. In contrast, Paschke et al. (2016) could prove an association between amygdala activity and a trial-by-trial arousal rating in a very similar emotion regulation task, which indicates that a post-hoc rating might not serve as a valid subjective evaluation of immediate arousal. Moreover, further psychophysiological measurements of emotional involvement (e.g., heart rate or skin conductance) were not used. Applying peripheral physiological techniques would have given us the opportunity to evaluate emotion regulation success with a further measurement (e.g., Masaoka, Hirasawa, Yamane, Hori, & Homma, 2003; Sakaki et al., 2016; Wallentin et al., 2011; Williams et al., 2001). Fixation of presented pictures was not controlled for via eye tracking. This means, we do not have an objective surveillance, whether participants fixated negative images. This could falsify brain activity during inspections of negative images. However, attendees were asked afterwards about their behavior during the experiment. All participants stated that they had followed the instructions and inspected every picture. Finally, we do not have the information if detachment is the participants preferred emotion regulation strategy. Maybe in their everyday life, some participants use other forms, for example, rationalization, another form of Reappraisal. While performing the task, they comply with the instructions, but would be more successful with their preferred strategy. However, we tried to address this by training of detachment before the scanner session.

## 6. Conclusion

Against our hypothesis, behavioral and neuronal emotion regulation success was not associated with individual differences in broad as well as narrow personality traits. One could assume that there is no association between personality traits and brain activity during emotion regulation. In many studies that consistently reported findings of activated and deactivated brain regions related to emotion regulation, associations between brain activity and personality traits were almost never mentioned.

We assume that there is a publication bias, that is, null findings were not published. fMRI studies with typically rather small samples are not adequately powered to detect at least medium-sized effects of *r* ≥ .30 at the nominal level of significance. In our sample with a substantially larger number of participants, we had a power of .80 to detect a correlation of nearly that size. Thus, our data suggest that associations between personality traits and emotion regulation success are of rather small size, if at all existing. Since emotion dysregulation plays a crucial role in the concept of several personality traits, further studies are needed to explore the probably more complex associations between emotion dysregulation, personality traits and, in the long run, well-being.

## Footnotes

1 Please note that parts of the following results will be included in Diers et al. (in revision). This concerns the neural main effects of emotion regulation, which we only described very briefly. These effects are briefly mentioned here, because in Diers et al. (in revision) the two independent samples will not be combined. We first wanted to check whether emotion regulation was successful in the combined sample, before we test our hypothesis regarding individual differences in emotion regulation.

## Supplementary material

For supplementary material accompanying this paper visit http://dx.doi.org/10.1017/pen.2019.11. click here to view supplementary material
