# Supplementary material for: Cognitive emotion regulation and personality: an analysis of individual differences in the neural and behavioral correlates of successful reappraisal
Source: Personal Neurosci. 2019 Nov 7;2:e11. doi: 10.1017/pen.2019.11 (PMC7219681; doi:10.1017/pen.2019.11)
Supplement: Supplementary file 1 [file S2513988619000117sup001.docx]

# Supplementary Material

## Supplementary Material 1

**Table S1**Results of Shaipiro-Wilk test for normal distribution of all predictors.

| **Variable** | **Mean** | **Std. Deviation** | **test statistic *W*** | **p-value** |
| --- | --- | --- | --- | --- |
| Behavioral emotion regulation success* | 16.27 | 33.6 | 0.96 | .026 |
| Neuronal emotion regulation success (left amygdala, sustained responses) | 0.33 | 1.14 | 0.99 | .51 |
| Neuronal emotion regulation success (left amygdala, transient responses)* | 4.26 | 8.66 | 0.94 | .001 |
| Neuronal emotion regulation success (right amygdala, sustained responses) | 0.26 | 0.97 | 0.92 | .274 |
| Neuronal emotion regulation success (right amygdala, transient responses) | 2.74 | 6.78 | 0.98 | .293 |
| Extend of the immediate aftereffect (left amygdala, sustained responses)* | 0.24 | 1.20 | 0.93 | < .001 |
| Extend of the immediate aftereffect (left amygdala, transient responses)* | 0.57 | 7.42 | 0.95 | .003 |
| Extend of the immediate aftereffect (right amygdala, sustained responses)* | 0.11 | 1.03 | 0.92 | < .001 |
| Extend of the immediate aftereffect (right amygdala, transient responses)* | 0.02 | 6.31 | 0.92 | < .001 |
| Neuroticism | 1.67 | 0.63 | 0.98 | .183 |
| Extraversion | 2.26 | 0.53 | 0.97 | .075 |
| Openness | 2.77 | 0.48 | 0.98 | .407 |
| Agreeableness | 2.72 | 0.48 | 0.99 | .615 |
| Conscientiousness | 2.72 | 0.57 | 0.98 | .211 |
| Positive Affect | 41.64 | 5.92 | 0.97 | .113 |
| Negative Affect* | 21.69 | 5.82 | 0.95 | .007 |
| ERQ-Suppression | 13.95 | 4.71 | 0.98 | .363 |
| ERQ-Reappraisal | 28.13 | 5.11 | 0.98 | .256 |
| *Note.* * Variable is not normal distributed. | | | | |

## Supplementary Material 2

**Table S2-1**

Neural correlates of intentional emotion regulation (sustained responses).

| **Region** | **H** | ***k*** | ***p_FWE_* /**  ***p_uncorr_*** | ***T*** | **x** | **y** | **z** |
| --- | --- | --- | --- | --- | --- | --- | --- |
| *Contrast: Negative Detach Stimulation > Negative Permit Stimulation* | | | | | | | |
| Angular gyrus / supramarginal gyrus / inferior parietal gyrus | r | 1007^+^ | < .001* | 8.32 | 52 | -50 | 34 |
| Superior frontal gyrus / supplementary motor area | r | 342^+^ | < .001* | 7.07 | 18 | 8 | 62 |
| Superior fronal gyrus / supplementary motor area / middle frontal gyrus | r |  | .001* | 5.88 | 16 | 18 | 52 |
| Superior frontal gyrus / superior frontal gyrus, medial / supplementary motor area | r |  | .005* | 5.43 | 14 | 26 | 50 |
| Middle frontal gyrus | r | 431^+^ | < .001* | 6.31 | 40 | 30 | 40 |
| Middle frontal gyrus | r |  | < .001* | 6.24 | 34 | 38 | 38 |
| Middle frontal gyrus | r |  | .017* | 5.11 | 38 | 16 | 46 |
| Cingulate gyrus, mid part | l/r | 256^+^ | < .001* | 6.21 | -4 | -26 | 26 |
| Cingulate gyrus, posterior part | r/l |  | .003* | 5.59 | 2 | -40 | 24 |
| Angular gyrus/ inferior parietal gyrus | l | 337^+^ | .001* | 5.29 | -40 | -52 | 36 |
| Supramarginal gyrus / angular gyrus / inferior parietal gyrus | l |  | .002* | 5.18 | -62 | -54 | 32 |
| Angular gyrus / inferior parietal gyrus | l |  | .006* | 5.40 | -50 | -58 | 38 |
| Precuneus / cuneus | r | 26^+^ | .013* | 5.18 | 12 | -68 | 36 |
| Inferior frontal gyrus, triangular / inferior frontal gyrus, orbital | l | 14^+^ | .018* | 5.08 | -54 | 34 | -4 |
| Inferior frontal gyrus, triangular / inferior frontal gyrus, orbital | r | 13^+^ | .021* | 5.04 | 48 | 38 | -8 |
| Precuneus / cuneus / superior parietal gyrus / superior occipital lobule | l | 20^+^ | .022* | 5.03 | -8 | -72 | 38 |
|  |  |  |  |  |  |  |  |
| *Contrast: Negative Permit Stimulation > Negative Detach Stimulation* | | | | | | | |
| Inferior occipital lobule / middle occipital lobule / calcarine sulcus | r | 1339^+^ | .000* | 7.71 | 28 | -96 | -2 |
| Calcarine sulcus | r |  | .001* | 5.84 | 14 | -100 | -2 |
| Inferior occipital lobule / inferior temporal gyrus | r |  | .013* | 5.18 | 50 | -74 | -10 |
| Inferior occipital lobule / middle occipital lobule / lingual gyrus | l | 1498^+^ | .000* | 6.84 | -24 | -96 | -8 |
| Gyrus rectus / medial orbitofrontal cortex | l/r | 209 | .000 | 4.20 | 0 | 34 | -18 |
| Gyrus rectus / olfactory cortex / medal orbitofrontal cortex | l/r |  | .001 | 3.36 | 4 | 22 | -16 |
| Amygdala / insula / temporal pole | l | 32 | .000 | 4.05 | -30 | 2 | -16 |
| Caudate nucleus / olfactory cortex / globus pallidus | l | 82 | .000 | 3.98 | -6 | 6 | -8 |
| Caudate nucleus / globus pallidus / olfactory cortex | l |  | .000 | 3.77 | 8 | 4 | -8 |
| Globus pallidus / hippocampus / amygdala | l |  | .000 | 3.49 | -10 | -2 | -10 |
| Caudate nucleus / anterior cingulate gyrus | l | 54 | .000 | 3.93 | -2 | 4 | 20 |
| Inferior temporal gyrus / fusiform gyrus | r | 42 | .000 | 3.66 | 46 | -44 | -14 |
| Medial orbitofrontal cortex / gyrus rectus | l/r | 16 | .001 | 3.40 | -4 | 48 | -14 |
| *Notes.* H - Hemisphere; l – left; r – right; *k* – cluster size > 10; x,y,z - respective coordinates of MNI template;* FWE corrected; ^+^ significant cluster on FWE level. | | | | | | | |

**
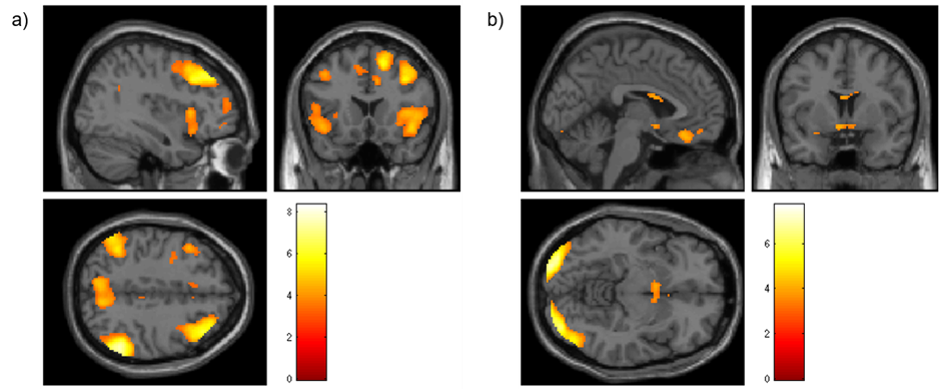
**

**Figure S2-1.** Whole brain results for two contrasts. a) Activation for the contrast: ‘Negative Detach Stimulation’ > ‘Negative Permit Stimulation’, *p* < .001 uncorr. Slices are at *x* = 38.1 (top left), *y* = 18.6 (top right), *z* = 41.4 (bottom left). b) Activation for the contrast: ‘Negative Permit Stimulation’ > ‘Negative Detach Stimulation’, *p* < .001 uncorr. Slices are at *x* = -2.6 (top left), *y* = 6.5 (top right), *z* = -10.6 (bottom left).

***
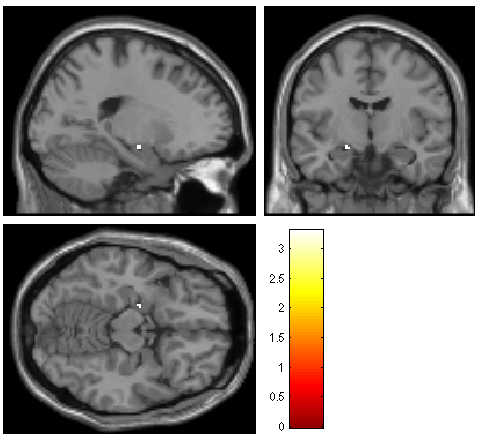
***

**Figure S2-2.** Amygdala activation for the contrast ‘Negative Permit Stimulation’ > ‘Negative Detach Stimulation’, *p* < .001 uncorr. Slices are at *x* = -19.4 (top left), *y* = -8.1 (top right), *z* = -14 (bottom left).

**Table S2*-2***

Neural correlates of intentional emotion regulation (transient responses).

| **Region** | **H** | ***k*** | ***p_FWE_* /**  ***p_uncorr_*** | ***T*** | **x** | **y** | **z** |
| --- | --- | --- | --- | --- | --- | --- | --- |
| *Contrast: Negative Detach Stimulation > Negative Permit Stimulation* | | | | | | | |
| Angular gyrus / inferior parietal gyrus / supramarginal gyrus | r | 462^+^ | .01* | 5.31 | 60 | -54 | 36 |
| Middle frontal gyrus | r | 67 | < .001 | 4.16 | 36 | 32 | 40 |
| Cingulate gyrus, mid part | r | 20 | < .001 | 3.98 | 8 | -22 | 30 |
| *Contrast: Negative Permit Stimulation > Negative Detach Stimulation* | | | | | | | |
| Gyrus rectus / medial orbitofrontal cortex / anterior cingulate cortex | l/r | 6200^+^ | < .001* | 6.79 | 4 | 30 | -16 |
| Insula / superior temporal pole / Amygdala / Putamen | l |  | .001* | 5.94 | -28 | 6 | -16 |
| Inferior frontal gyrus, pars triangularis and pars opercularis / Insula | l |  | .005* | 5.55 | -30 | 16 | 22 |
| Inferior occipital gyrus / fusiform face area / middle occipital gyrus | r | 15016^+^ | .003* | 5.69 | 34 | -86 | -8 |
| Superior occipital gyrus / middle occipital gyrus / Cuneus / superior parietal lobule / Precuneus | r |  | .003* | 5.67 | 26 | -74 | 40 |
| Cerebellum | r |  | .007* | 5.41 | -22 | -34 | -40 |
| Inferior frontal gyrus, pars orbitalis / posterior and anterior orbitofrontal cortex / Insula | r | 369^+^ | .05* | 4.84 | 30 | 34 | -6 |
| Middle frontal gyrus / inferior frontal gyrus, pars triangularis | r |  | < .001 | 4.36 | 50 | 46 | 4 |
| Inferior frontal gyrus, pars triangularis / middle frontal gyurs | r |  | < .001 | 4.02 | 54 | 40 | 10 |
| Thalamus | l/r | 228 | < .001 | 4.80 | 2 | -8 | 4 |
| Insula / middle frontal gyrus | r | 856^+^ | < .001 | 4.79 | 28 | 26 | 16 |
| Inferior frontal gyrus, pars opercularis and pars triangularis / middle frontal gyurs / precentral gyurs | r |  | < .001 | 4.72 | 42 | 14 | 28 |
| Inferior frontal gyrus, pars triangularis / Insula | r |  | < .001 | 4.06 | 30 | 18 | 20 |
| Caudate nucleus / anterior cingulate cortex | l | 231 | < .001 | 4.60 | -4 | 12 | 16 |
| Olfactory cortex / Insula / Putamen / posterior orbitofrontal cortex | r | 846^+^ | < .001 | 4.57 | 24 | 12 | -16 |
| Hippocamups / Amygdala | r |  | < .001 | 4.39 | 22 | -10 | -12 |
| Parahippocampal gyrus / Amygdala / olfactory cortex / superior temporal pole | r |  | < .001 | 4.38 | 22 | 6 | -22 |
| Inferior frontal gyrus, pars opercularis / caudate nucleus | r | 120 | < .001 | 4.42 | 24 | 8 | 30 |
| Midcingulate area | l/r | 288 | < .001 | 4.41 | 0 | -6 | 42 |
| Midcingulate area / supplementary motor area | r |  | < .001 | 4.25 | 10 | -14 | 44 |
| Midcingulate area / anterior cingulate cortex | l/r |  | < .001 | 3.38 | -4 | 8 | 36 |
| Superior parietal lobule / inferior parietal lobule | l | 223 | < .001 | 4.08 | -28 | -62 | 54 |
| Superior parietal lobule / inferior parietal lobule | l |  | < .001 | 4.04 | -30 | -54 | 56 |
| Inferior parietal lobule / superior parietal lobule | l |  | < .001 | 3.94 | -26 | -52 | 48 |
| Postcentral gyrus / rolandic operculum / supramarginal gyrus | r | 144 | < .001 | 4.02 | 46 | -14 | 28 |
| Supramarginal gyrus / rolandic operculum / superior temporal gyrus | r |  | < .001 | 3.70 | 56 | -24 | 24 |
| Precentral gyrus / paracentral lobule / postcentral gyrus | l | 130 | < .001 | 4.01 | -18 | -22 | 64 |
| Paracentral lobule / precentral gyrus | l |  | < .001 | 3.88 | -14 | -26 | 56 |
| Postcentral gyrus / superior parietal lobule | l |  | < .001 | 3.78 | -28 | -40 | 60 |
| Precuneus / midcingulate area | l | 203 | < .001 | 4.01 | -8 | -46 | 48 |
| Precuneus / midcingulate area | l |  | < .001 | 3.85 | -14 | -46 | 40 |
| Supplementary motor area | l/r | 172 | < .001 | 3.91 | 4 | -2 | 66 |
| Supplementary motor area | l/r |  | < .001 | 3.85 | -4 | -12 | 60 |
| Lingula gyrus / lobule III of vermis / Thalamus | r | 107 | < .001 | 3.75 | 4 | -32 | -4 |
| Lobule IV,V of vermis / Cerebelum / lingual gyrus | l |  | < .001 | 3.44 | -2 | -44 | -2 |
| Paracentral lobule / Precuneus / midcingulate area / postcentral gyrus / supplementary motor area | r | 24 | < .001 | 3.68 | 10 | -34 | 56 |
| Superior frontal gyrus / medial frontal gyrus | l | 23 | < .001 | 3.52 | -16 | 22 | 40 |
| Supramarginal gyrus / superior temporal gyrus / rolandic operculum | r | 11 | < .001 | 3.50 | 44 | -36 | 24 |
| Postcentral gyrus / inferior parietal lobule | l | 14 | < .001 | 3.48 | -44 | -30 | 46 |
| Thalamus / caudate nucleus | r | 16 | < .001 | 3.47 | 20 | -20 | 18 |
| Supramarginal gyrus / inferior temporal gyrus | l | 14 | < .001 | 3.41 | -62 | -30 | 24 |
| *Notes.* H - Hemisphere; l – left; r – right; *k* – cluster size; *x,y,z* - respective coordinates of MNI template;* FWE corrected; ^+^ significant cluster on FWE level. | | | | | | | |


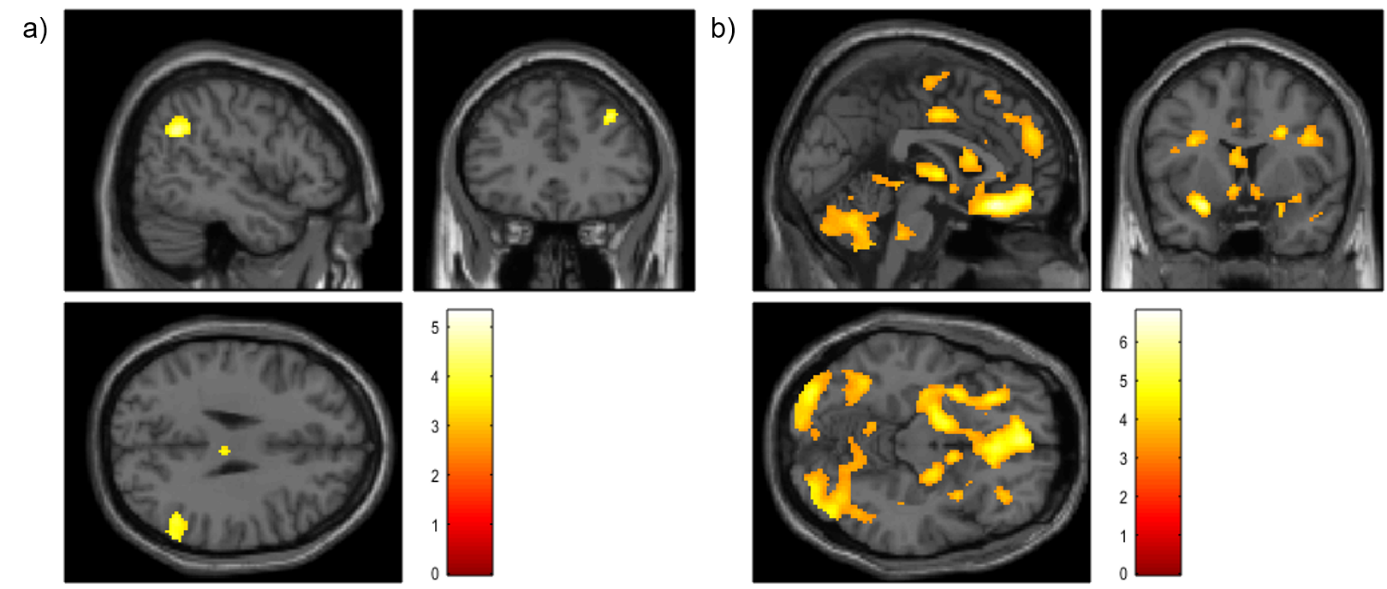


**Figure S2-3.** Whole brain results for two contrasts using stick functions. a) Activation for the contrast: ‘Negative Detach Stimulation’ > ‘Negative Permit Stimulation’, *p* < .001 uncorr. Slices are at *x* = 49.4 (top left), K = 31.5 (top right), *z* = 28.8 (bottom left). b) Activation for the contrast: ‘Negative Permit Stimulation’ > ‘Negative Detach Stimulation’, *p* < .001 uncorr. Slices are at *x* = 0 (top left), *y* = 7.4 (top right), *z* = 11.7 (bottom left).


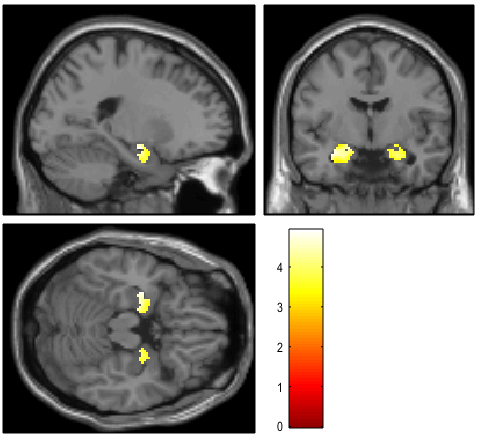


**Figure S2-4.** Amygdala activation for the contrast ‘Negative Permit Stimulation’ > ‘Negative Detach Stimulation’, *p* < .001 uncorr. Slices are at *x* = -20.8 (top left), *y* = -5.5 (top right), *z* = -18.6 (bottom left).

## Supplementary Material 3

**Table S3-1**

Correlations between neuronal (sustained responses) and behavioral emotion regulation success.

| Variables | 1 | 2 | 3 |
| --- | --- | --- | --- |
| 1. Arousal Rating | - |  |  |
| 2. left amygdala activity | - .04 | - |  |
| 3. right amygdala activity | .07 | .81*** | - |
| *Note.* * *p* < .05. ** *p* < .01. *** *p* < .001 | | | |

**Table S3-2**

Correlations between neuronal (transient responses) and behavioral emotion regulation success.

| Variables | 1 | 2 | 3 |
| --- | --- | --- | --- |
| 1. Arousal Rating | - |  |  |
| 2. left amygdala activity | - .06 | - |  |
| 3. right amygdala activity | - .02 | .79*** | - |
| *Note.* * *p* < .05. ** *p* < .01. *** *p* < .001 | | | |

## Supplementary Material 4

**Table S4**

Reverse task-rest interaction effects (unmasked) for sustained responses.

| **Region** | **H** | ***k*** | ***p_FWE_* /**  ***p_uncorr_*** | ***T*** | **x** | **y** | **z** |
| --- | --- | --- | --- | --- | --- | --- | --- |
| *Contrast: (Negative Permit Stimulation > Negative Detach Stimulation) > (Negative Detach Relaxation > Negative Permit Relaxation)* | | | | | | | |
| Lingual gyrus / calcarine sulcus / inferior occipital lobule | l | 14890^+^ | .000* | 6.93 | -14 | -92 | -14 |
| Inferior occipital lobule / lingual gyrus | r |  | .000* | 6.87 | 30 | -94 | -8 |
| Calcarine sulcus / lingual gyrus | r |  | .000* | 6.69 | 14 | -100 | -2 |
| Precentral gyrus/ superior frontal gyrus | r | 60 | .013* | 5.17 | 34 | -14 | 66 |
| Midcingulate area / supplementary motor area | l/r | 394^+^ | .000 | 4.44 | -4 | -10 | 46 |
| Midcingulate area / supplementary motor area | l/r |  | .000 | 4.22 | 6 | -12 | 48 |
| Midcingulate area / supplementary motor area | l |  | .000 | 3.72 | -14 | -18 | 46 |
| Hippocampus / parahippocampal gyrus | l | 415^+^ | .000 | 4.28 | -24 | -16 | -18 |
| Amygdala / insula / superior temporal pole | l |  | .000 | 3.63 | -30 | 2 | -16 |
| Middle temporal gyrus / superior temporal pole / inferior temporal gyrus | r | 41 | .000 | 4.26 | 46 | -6 | -20 |
| Cerebelum | l | 48 | .000 | 4.21 | -12 | -38 | -40 |
| Caudate nucleus | l | 190 | .000 | 4.12 | -20 | 16 | 22 |
| Anterior cingulate gyrus / caudate nucleus | l |  | .000 | 4.00 | -6 | 8 | 22 |
| Anterior cingulate gyrus | l/r |  | .000 | 3.58 | 4 | 8 | 20 |
| Vermis / cerebelum | l/r | 102 | .000 | 4.06 | 2 | -54 | -38 |
| Midcingulate area / paracentral lobule | l | 80 | .000 | 4.01 | -16 | -34 | 46 |
| Medial frontal gyrus / superior frontal gyrus | l | 52 | .000 | 3.71 | -4 | 56 | 38 |
| Medial frontal gyrus | l | 28 | .000 | 3.69 | 0 | 34 | 58 |
| Midcingulate area / paracentral lobule / postcentral gyrus | r | 19 | .000 | 3.67 | 20 | -34 | 46 |
| Inferior temporal gyrus / fusiform gyrus | r | 2 | .000 | 3.61 | 50 | -26 | -26 |
| Cerebelum | r | 26 | .000 | 3.58 | 14 | -46 | -40 |
| Superior temporal gyrus / Rolandic operculum | r | 32 | .000 | 3.56 | 64 | -2 | 4 |
| Insula / Rolandic operculum | l | 17 | .000 | 3.55 | -42 | -2 | 6 |
| Thalamus / hippocampus | l | 6 | .000 | 3.48 | -22 | -32 | 8 |
| Precuneus / midcingulate area | l | 18 | .000 | 3.44 | -10 | -46 | 44 |
| Cerebelum / vermis | l | 30 | .000 | 3.43 | -4 | -76 | -38 |
| Superior temporal gyrus / supramarginal gyrus | l | 13 | .001 | 3.37 | -62 | -30 | 20 |
| Cerebelum / vermis | r | 11 | .001 | 3.29 | 4 | -32 | -18 |
| *Notes.* H - Hemisphere; *k* – cluster size > 10; x,y,z - respective coordinates of MNI template;* FWE corrected; ^+^ significant cluster on FWE level. | | | | | | | |

## Supplementary Material 5

**Table S5-1**

Reverse task-rest interaction effects (inclusively masked) for sustained responses.

| **Region** | **H** | ***k*** | ***p_FWE_* /**  ***p_uncorr_*** | ***T*** | **x** | **y** | **z** |
| --- | --- | --- | --- | --- | --- | --- | --- |
| *Contrast: (Negative Permit Stimulation > Negative Detach Stimulation) > (Negative Detach Relaxation > Negative Permit Relaxation)* | | | | | | | |
| Lingual gyrus / calcarine sulcus / inferior occipital lobule / cerebellum | l | 179^+^ | .000* | 6.93 | -14 | -92 | -14 |
| Inferior occipital lobule / lingual gyrus | l |  | .000* | 6.42 | -34 | -90 | -16 |
| Inferior occipital lobule / fusiform gyrus / cerebellum | l |  | .002* | 5.68 | -44 | -80 | -14 |
| Inferior occipital lobule / lingual gyrus / fusiform gyrus | r | 179^+^ | .000* | 6.84 | 28 | -92 | -10 |
| Inferior occipital lobule / inferior temporal gyrus/ fusiform gyrus | r |  | .001* | 6.02 | 44 | -78 | -12 |
| Inferior temporal gyrus / inferior occipital lobule / fusiform gyrus | r |  | .001* | 5.81 | 50 | -68 | -14 |
| Middle occipital gyrus / calcarine sulcus / superior occipital gyrus / cuneus | r | 93^+^ | .000* | 6.22 | 24 | -94 | 6 |
| Calcarine sulcus / cuneus / superior occipital gyrus | r |  | .001* | 5.88 | 14 | -100 | 2 |
| Middle occipital gyrus | l | 72^+^ | .001* | 6.05 | -28 | -90 | 10 |
| Calcarine sulcus/ lingual gyrus / cerebelum | l | 1^+^ | .002* | 5.74 | -2 | -86 | -14 |
| *Notes.* H - Hemisphere; l – left; r – right; *k* – cluster size; x,y,z - respective coordinates of MNI template;* FWE corrected; ^+^ significant cluster on FWE level. | | | | | | | |

**Table S5-2**

Reverse task-rest interaction effects (inclusively masked) for transient responses.

| **Region** | **H** | ***k*** | ***p_FWE_*** | ***T*** | **x** | **y** | **z** |
| --- | --- | --- | --- | --- | --- | --- | --- |
| *Contrast: (Negative Permit Stimulation > Negative Detach Stimulation) > (Negative Detach Relaxation > Negative Permit Relaxation)* | | | | | | | |
| Superior occipital gyrus / middle occipital gyrus / cuneus | r | 173^+^ | < .001 | 7.25 | 24 | -82 | 24 |
| Calcarine sulcus / cuneus / superior occipital gyurs | r |  | < .001 | 5.40 | 18 | -90 | 6 |
| Cerebelum | l | 387^+^ | < .001 | 6.82 | -32 | -78 | -40 |
| Cerebelum | l |  | < .001 | 6.26 | -42 | -70 | -32 |
| Cerebelum / lingual gyrus | l |  | < .001 | 5.52 | -20 | -68 | -14 |
| Superior occipital gyrus / middle occipital gyrus / cuneus | l | 95^+^ | < .001 | 6.74 | -22 | -80 | 28 |
| Middle occipital gyrus / middle temporal gyrus | l | 26^+^ | < .001 | 6.65 | -42 | -70 | 8 |
| Cerebelum / lingural gyrus / fusiform face area | l | 116^+^ | < .001 | 6.39 | -24 | -86 | -20 |
| Calcarine sulcus / lingual gyrus | l |  | < .001 | 5.98 | -8 | -94 | -12 |
| Cerebelum | l |  | < .001 | 5.68 | -20 | -74 | -28 |
| Superior parietal lobule / inferior parietal lobule | l | 45^+^ | < .001 | 6.34 | -32 | -58 | 54 |
| Cerebelum | r | 15^+^ | < .001 | 6.25 | 28 | -76 | -44 |
| Cerebelum | l | 5^+^ | < .001 | 6.22 | -18 | -84 | -36 |
| Calcarine sulcus / lingual gyrus | l/r | 3^+^ | < .001 | 6.18 | 4 | -74 | 6 |
| Superior parietal lobule / angular gyrus / inferior parietal lobule | r | 15^+^ | < .001 | 6.05 | 30 | -60 | 56 |
| Superior and inferior parietal lobule / posterior orbitofrontal cortex | r |  | .017 | 5.14 | 34 | -52 | 58 |
| Insula / inferior frontal gyrus, pars triangularis / middle frontal gyrus | r | 26^+^ | .001 | 6.04 | 32 | 36 | 2 |
| anterior orbitofrontal cortex / inferio frontal gyrus, pars orbitalis / posterior orbitofrontal cortex | r |  | .001 | 5.82 | 34 | 38 | -14 |
| Superior frontal gyrus, medial part | l | 61^+^ | .001 | 5.89 | -4 | 38 | 38 |
| Inferior frontal gyrus, pars triangularis and orbitalis / middle frontal gyrus | l | 65^+^ | .001 | 5.89 | -46 | 44 | 0 |
| Inferior frontal gyrus, pars opercularis and triangularis / Insula | l | 25^+^ | .001 | 5.89 | -36 | 12 | 22 |
| Calcarine sulcus / lingual gyrus | r | 12^+^ | .002 | 5.76 | 10 | -66 | 10 |
| Cerebelum | l | 16^+^ | .002 | 5.74 | -8 | -80 | -30 |
| Inferior frontal gyrus, pars triangularis | r | 18^+^ | .002 | 5.69 | 32 | 26 | 16 |
| Cerebelum | r | 10^+^ | .007 | 5.39 | 46 | -66 | -36 |
| Superior frontal gyrus, medial part | l/r | 31^+^ | .007 | 5.39 | 0 | 32 | 54 |
| Fusiform face area / lingual gyrus | r | 14^+^ | .011 | 5.27 | 28 | -72 | -12 |
| *Notes.* H - Hemisphere; *k* – cluster size > 10; x,y,z - respective coordinates of MNI template; ^+^ significant cluster on FWE level. | | | | | | | |

## Supplementary Material 6

**Table S6-1**

Summary of Hierarchical Regression Analysis for variables predicting neuronal emotion regulation success in right amygdala (sustained responses).

| **Variable** | **β** | ***t*** | ***p*** | ***R*** | ***R²*** | ***∆R²*** |
| --- | --- | --- | --- | --- | --- | --- |
| Step 1 | | | | 0.14 | 0.020 | 0.020 |
| Sample | - .02 | - 0.21 | .831 |  |  |  |
| Step 2 | | | | 0.24 | 0.056 | 0.036 |
| Sample | - .18 | - 1.45 | .153 |  |  |  |
| Negative Affect | - .10 | - 0.66 | .514 |  |  |  |
| Positive Affect | - .03 | - 0.16 | .877 |  |  |  |
| Neuroticism | .00 | - 0.01 | .991 |  |  |  |
| Extraversion | - .16 | - 0.82 | .413 |  |  |  |
| ERQ – Suppression | .02 | 0.13 | .897 |  |  |  |
| ERQ – Reappraisal | .17 | 1.25 | .217 |  |  |  |
| Step 3 | | | | 0.25 | 0.061 | 0.005 |
| Sample | - .17 | - 1.32 | .192 |  |  |  |
| Positive Affect | - .08 | - 0.51 | .614 |  |  |  |
| Negative Affect | - .06 | - 0.32 | .748 |  |  |  |
| Neuroticism | .00 | - 0.01 | .991 |  |  |  |
| Extraversion | - .16 | - 0.81 | .423 |  |  |  |
| ERQ – Suppression | .04 | 0.27 | .790 |  |  |  |
| ERQ – Reappraisal | .18 | 1.24 | .219 |  |  |  |
| Openness | - .03 | - 0.23 | .822 |  |  |  |
| Agreeableness | .04 | 0.28 | .781 |  |  |  |
| Conscientiousness | .06 | 0.42 | .676 |  |  |  |
| *Note. N* = 76. | | | | | | |

**Table S6-2**

Summary of Hierarchical Regression Analysis for variables predicting neuronal emotion regulation success in left amygdala activity (transient responses).

| **Variable** | **β** | ***t*** | ***p*** | ***R*** | ***R²*** | ***∆R²*** |
| --- | --- | --- | --- | --- | --- | --- |
| Step 1 | | | | 0.04 | 0.002 | 0.002 |
| Sample | - .05 | - 0.43 | .667 |  |  |  |
| Step 2 | | | | 0.24 | 0.060 | 0.058 |
| Sample | - .12 | - 0.94 | .348 |  |  |  |
| Negative Affect | .09 | 0.61 | .546 |  |  |  |
| Positive Affect | - .16 | - 0.94 | .353 |  |  |  |
| Neuroticism | .08 | 0.46 | .650 |  |  |  |
| Extraversion | .11 | 0.56 | .578 |  |  |  |
| ERQ – Suppression | - .01 | - 0.09 | .930 |  |  |  |
| ERQ – Reappraisal | .02 | 0.15 | .879 |  |  |  |
| Step 3 | | | | 0.33 | 0.112 | 0.052 |
| Sample | - .10 | - 0.79 | .430 |  |  |  |
| Positive Affect | .13 | 0.81 | .422 |  |  |  |
| Negative Affect | - .22 | - 1.18 | .244 |  |  |  |
| Neuroticism | .13 | 0.70 | .484 |  |  |  |
| Extraversion | .13 | 0.68 | .501 |  |  |  |
| ERQ – Suppression | .00 | - 0.02 | .981 |  |  |  |
| ERQ – Reappraisal | .12 | 0.86 | .391 |  |  |  |
| Openness | - .12 | - 0.92 | .361 |  |  |  |
| Agreeableness | - .10 | - 0.68 | .500 |  |  |  |
| Conscientiousness | .21 | 1.40 | .167 |  |  |  |
| *Note. N* = 76. | | | | | | |

**Table S6-3**

Summary of Hierarchical Regression Analysis for variables predicting neuronal emotion regulation success in right amygdala activity (transient responses).

| **Variable** | **β** | ***t*** | ***p*** | ***R*** | ***R²*** | ***∆R²*** |
| --- | --- | --- | --- | --- | --- | --- |
| Step 1 | | | | 0.06 | 0.004 | 0.004 |
| Sample | .06 | 0.57 | .571 |  |  |  |
| Step 2 | | | | 0.15 | 0.023 | 0,019 |
| Sample | .01 | 0.08 | .938 |  |  |  |
| Negative Affect | - .03 | - 0.17 | .864 |  |  |  |
| Positive Affect | - .07 | - 0.41 | .68 |  |  |  |
| Neuroticism | .12 | 0.63 | .533 |  |  |  |
| Extraversion | .10 | 0.49 | .627 |  |  |  |
| ERQ – Suppression | - .06 | - 0.45 | .657 |  |  |  |
| ERQ – Reappraisal | - .05 | - 0.40 | .690 |  |  |  |
| Step 3 | | | | 0.31 | 0.098 | 0,075 |
| Sample | .03 | 0.27 | .788 |  |  |  |
| Positive Affect | .03 | 0.17 | .863 |  |  |  |
| Negative Affect | - .17 | - 0.88 | .380 |  |  |  |
| Neuroticism | .16 | 0.85 | .397 |  |  |  |
| Extraversion | .11 | 0.60 | .553 |  |  |  |
| ERQ – Suppression | - .03 | - 0.20 | .844 |  |  |  |
| ERQ – Reappraisal | .06 | 0.41 | .683 |  |  |  |
| Openness | - .15 | - 1.18 | .241 |  |  |  |
| Agreeableness | - .04 | - 0.26 | .794 |  |  |  |
| Conscientiousness | .26 | 1.74 | .087 |  |  |  |
| *Note. N* = 76. | | | | | | |

## Supplementary Material 7


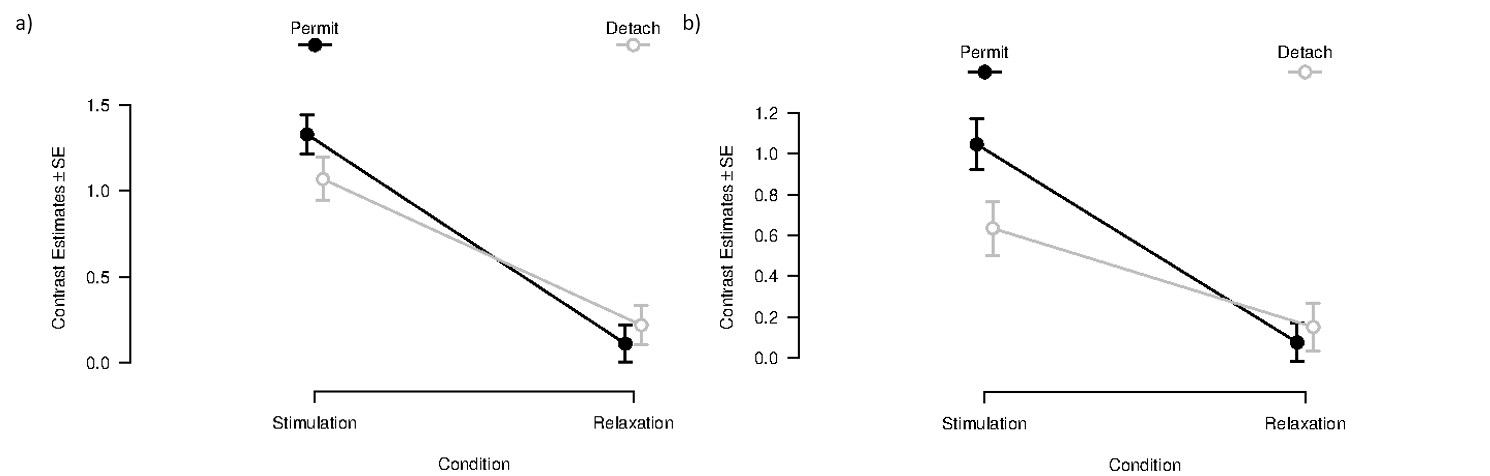


**Figure S7-1.** Contrast estimates for sustained responses in a functionally defined ROI in the right amygdala (*x* = 22, *y* = -6, *z* = -12). Left (a): Beta-values during and after inspection of negative images for the conditions ‘permit’ (black line) and ‘detach’ (gray line); error bars indicate *SD*. Right (b): Beta-values during and after inspection of neutral images for the conditions ‘permit’ (black line) and ‘detach’ (gray line); error bars indicate *SD*.


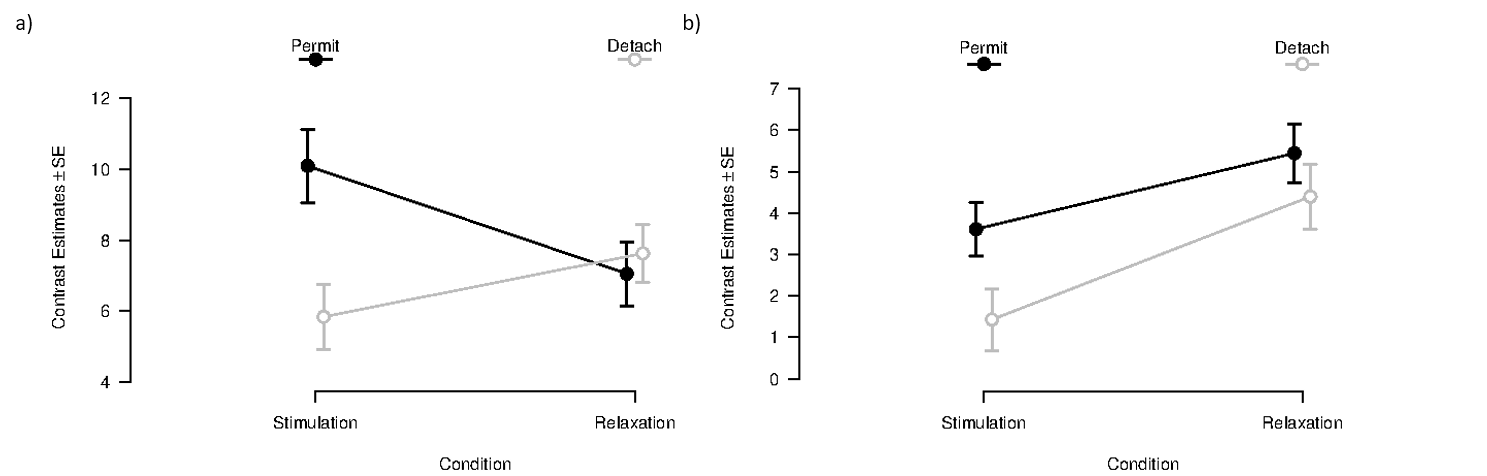


**Figure S7-2.** Contrast estimates for transient responses in a functionally defined ROI in the left amygdala (*x* = -18, *y* = -6, *z* = -14). Left (a): Beta-values during and after inspection of negative images for the conditions ‘permit’ (black line) and ‘detach’ (gray line); error bars indicate *SD*. Right (b): Beta-values during and after inspection of neutral images for the conditions ‘permit’ (black line) and ‘detach’ (gray line); error bars indicate *SD*.


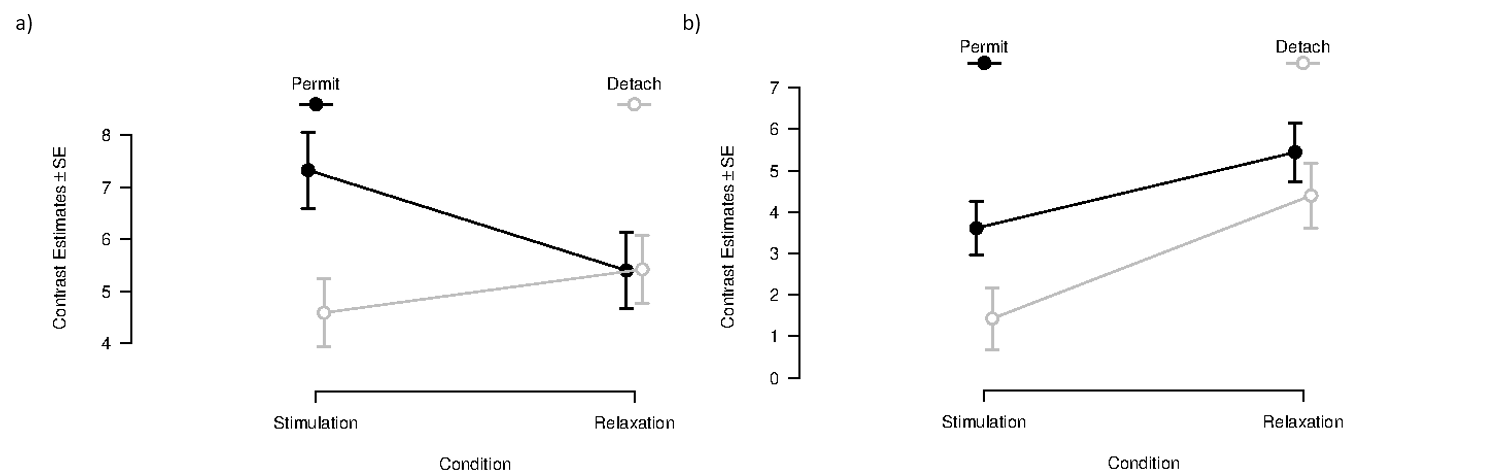


**Figure S7-3.** Contrast estimates for transient responses in a functionally defined ROI in the right amygdala (*x* = 22, *y* = -6, *z* = -12). Left (a): Beta-values during and after inspection of negative images for the conditions ‘permit’ (black line) and ‘detach’ (gray line); error bars indicate *SD*. Right (b): Beta-values during and after inspection of neutral images for the conditions ‘permit’ (black line) and ‘detach’ (gray line); error bars indicate *SD*.

## Supplementary Material 8


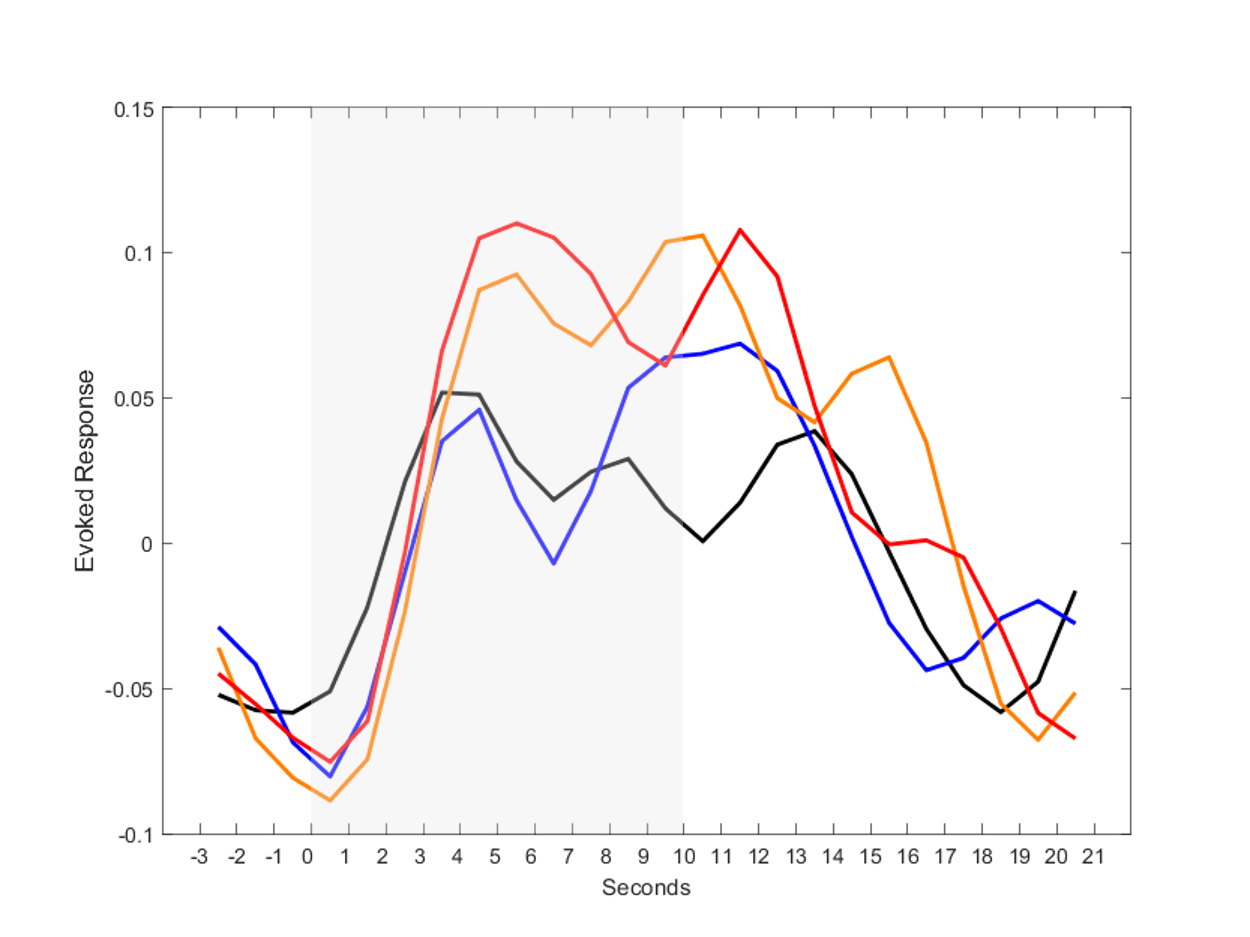


**Figure S8.** Activation time-courses in the right amygdala for different conditions. Peak voxel was *x* = 22, *y* = -6, *z* = -14. Color indicates different conditions: black – ‘neutral detach’, blue – ‘neutral permit’, orange – ‘negative detach’, red – ‘negative permit’. Shaded area indicates phase of picture presentation.

## Supplementary Material 9

**Table S9-1**

Summary of Partial Correlation Analysis between of the immediate aftereffect in the amygdala (left and right) (sustained responses) and personality traits.

| Variables | 1 | 2 | 3 | 4 | 5 | 6 | 7 | 9 | 10 | 11 | 12 |
| --- | --- | --- | --- | --- | --- | --- | --- | --- | --- | --- | --- |
| 1. Neuroticism | - |  |  |  |  |  |  |  |  |  |  |
| 2. Extraversion | - .55*** | - |  |  |  |  |  |  |  |  |  |
| 3. Openness | --.02 | .09 | - |  |  |  |  |  |  |  |  |
| 4. Agreeableness | - .15 | .40*** | .01 | - |  |  |  |  |  |  |  |
| 5. Conscientiousness | - .36** | .31** | - .17 | .07 | - |  |  |  |  |  |  |
| 6. Positive Affect | - .60*** | .62*** | .09 | .29** | .42*** | - |  |  |  |  |  |
| 7. Negative Affect | .60*** | - .37** | .10 | - .25* | - .22* | - .26* | - |  |  |  |  |
| 8. ERQ-Suppression | .17 | - .47*** | - .09 | - .41*** | - .20 | - .18 | .22* | - |  |  |  |
| 9. ERQ-Reappraisal | -.29** | .41*** | .23* | .40*** | - .02 | .29** | - .18 | - .33* | - |  |  |
| 10. left amygdala | .07 | -.03 | - .05 | - .06 | .06 | .02 | - .03 | .01 | - .13 | - |  |
| 11. right amygdala | - .06 | .06 | - .07 | .09 | .05 | .14 | - .16 | .14 | - .12 | .88*** | - |
| *M* | 1.67 | 2.25 | 2.78 | 2.71 | 2.71 | 41.61 | 21.69 | 13.95 | 28.13 | 0.20 | 0.10 |
| *SD* | 0.63 | 0.53 | 0.48 | 0.48 | 0.57 | 6.01 | 5.87 | 4.71 | 5.11 | 1.21 | 1.10 |
| *Note. N* = 75. * *p* < .05. ** *p* < .01. *** *p* < .001 | | | | | | | | | | | |

**Table S9-2**

Summary of Partial Correlation Analysis between the immediate aftereffect in the amygdala (left and right) (transient responses) and personality traits.

| Variables | 1 | 2 | 3 | 4 | 5 | 6 | 7 | 8 | 9 | 10 | 11 | 12 |
| --- | --- | --- | --- | --- | --- | --- | --- | --- | --- | --- | --- | --- |
| 1. Neuroticism | - |  |  |  |  |  |  |  |  |  |  |  |
| 2. Extraversion | - .55*** | - |  |  |  |  |  |  |  |  |  |  |
| 3. Openness | .02 | .09 | - |  |  |  |  |  |  |  |  |  |
| 4. Agreeableness | - .15 | .40*** | .01 | - |  |  |  |  |  |  |  |  |
| 5. Conscientiousness | - .36** | .31** | - .17 | .07 | - |  |  |  |  |  |  |  |
| 6. Positive Affect | - .60*** | .62*** | .09 | .29** | .42*** | - |  |  |  |  |  |  |
| 7. Negative Affect | .60*** | - .37** | .10 | - .25* | - .22* | - .26* | - |  |  |  |  |  |
| 8. ERQ-Suppression | .17 | - .47*** | - .09 | - .41*** | - .20 | - .18 | .22* | .38*** | - |  |  |  |
| 9. ERQ-Reappraisal | - .29** | .41*** | .23* | .40*** | - .02 | .29** | - .18 | - .25* | - .33** | - |  |  |
| 10. left amygdala | .08 | - .01 | .06 | - .06 | - .03 | - .03 | .10 | .00 | .13 | - .13 | - |  |
| 11. right amygdala | .08 | -.01 | .13 | - .12 | - .06 | - .13 | .08 | .03 | .13 | - .13 | . 80 | - |
| *M* | 1.67 | 2.25 | 2.78 | 2.71 | 2.71 | 41.61 | 21.69 | 2.70 | 13.95 | 28.13 | 0.57 | 0.02 |
| *SD* | 0.63 | 0.53 | 0.48 | 0.48 | 0.57 | 6.01 | 5.87 | 0.74 | 4.71 | 5.11 | 7.42 | 6.31 |
| *Note. N* = 75. * *p* < .05. ** *p* < .01. *** *p* < .001 | | | | | | | | | | |  |  |
